# *S6K1* amplification confers innate resistance to CDK4/6 inhibitors through activating c-Myc pathway in patients with estrogen receptor-positive breast cancer

**DOI:** 10.1186/s12943-022-01642-5

**Published:** 2022-08-30

**Authors:** Hongnan Mo, Xuefeng Liu, Yu Xue, Hongyan Chen, Shichao Guo, Zhangfu Li, Shuang Wang, Caiming Li, Jiashu Han, Ming Fu, Yongmei Song, Dan Li, Fei Ma

**Affiliations:** 1 Department of Medical Oncology, National Cancer Center/National Clinical Research Center for Cancer/Cancer Hospital, Chinese Academy of Medical Sciences and Peking Union Medical College, No.17 Panjiayuan Nanli, Chaoyang District, Beijing, 100021 China; 2 Institute of Cancer Stem Cell, Dalian Medical University, Dalian, China; 3 State Key Laboratory of Molecular Oncology, National Cancer Center/National Clinical Research Center for Cancer/Cancer Hospital, Chinese Academy of Medical Sciences and Peking Union Medical College, No.17 Panjiayuan Nanli, Chaoyang District, Beijing, 100021 China; 4 State Key Laboratory of Microbial Resources, Institute of Microbiology, Chinese Academy of Sciences, Beijing, China

**Keywords:** Breast cancer, CDK4/6 inhibitors, Drug resistance, Circulating tumour DNA, S6K1

## Abstract

**Background:**

CDK4/6 inhibitors combined with endocrine therapy has become the preferred treatment approach for patients with estrogen receptor-positive metastatic breast cancer. However, the predictive biomarkers and mechanisms of innate resistance to CDK4/6 inhibitors remain largely unknown. We sought to elucidate the molecular hallmarks and therapeutically actionable features of patients with resistance to CDK4/6 inhibitors.

**Methods:**

A total of 36 patients received palbociclib and endocrine therapy were included in this study as the discovery cohort. Next-generation sequencing of circulating tumour DNA in these patients was performed to evaluate somatic alterations associated with innate resistance to palbociclib. Then the candidate biomarker was validated in another independent cohort of 104 patients and publicly available datasets. The resistance was verified in parental MCF-7 and T47D cells, as well as their derivatives with small interfering RNA transfection and lentivirus infection. The relevant mechanism was examined by RNA sequencing, chromatin immunoprecipitation and luciferase assay. Patient-derived organoid and patient-derived xenografts studies were utilized to evaluated the antitumor activity of rational combinations.

**Results:**

In the discovery cohort, *S6K1* amplification (3/35, 9%) was identified as an important reason for innate resistance to CDK4/6 inhibitors. In the independent cohort, S6K1 was overexpressed in 15/104 (14%) patients. In those who had received palbociclib treatment, patients with high-expressed S6K1 had significantly worse progression free survival than those with low S6K1 expression (hazard ratio = 3.0, *P* = 0.0072). Meta-analysis of public data revealed that patients with *S6K1* amplification accounted for 12% of breast cancers. Breast cancer patients with high *S6K1* expression had significantly worse relapse-free survival (hazard ratio = 1.31, *P* < 0.0001). In breast cancer cells, S6K1 overexpression, caused by gene amplification, was sufficient to promote resistance to palbociclib. Mechanistically, S6K1 overexpression increased the expression levels of G1/S transition-related proteins and the phosphorylation of Rb, mainly through the activation of c-Myc pathway. Notably, this resistance could be abrogated by the addition of mTOR inhibitor, which blocked the upstream of S6K1, in vitro and in vivo.

**Conclusions:**

*S6K1* amplification is an important mechanism of innate resistance to palbociclib in breast cancers. Breast cancers with *S6K1* amplification could be considered for combinations of CDK4/6 and S6K1 antagonists.

**Supplementary Information:**

The online version contains supplementary material available at 10.1186/s12943-022-01642-5.

## Background

Breast cancer is the most common malignant tumour in women worldwide, with 272,4000 of new cases per year in China [1–3]. Estrogen receptor-positive (ER^+^) human epidermal growth factor receptor 2-negative (HER2^−^) breast cancer is the most common subtype, accounting for more than 60% of the metastatic breast cancers (MBCs) [4]. Cyclin-dependent kinase 4 and 6 (CDK4/6) inhibitors, including palbociclib, ribociclib, and abemaciclib, have been shown to significantly improve progression-free survival (PFS) and overall survival (OS) in patients with ER^+^HER2^−^ MBC [5–8]. Thus, CDK4/6 inhibitors in combination with endocrine therapy has become the most important treatment option for patients with ER^+^HER2^−^ MBC.

However, the prevalence of drug resistance reduces the effect of CDK4/6 inhibitor treatment in patients with ER^+^HER2^−^ MBC. Some patients do not derive any benefit from CDK4/6 inhibitors and often switch to other therapies within 3 months—defined as innate resistance. In order to select more appropriate regimen and avoid the adverse events of palbociclib in these patients, it is critical to identify biomarkers that can predict innate resistance to palbociclib. Unfortunately, the biomarker analysis of several large-scale phase III clinical trials failed to establish constant biomarkers to predict the efficacy of the combined use of palbociclib with endocrine therapy, mainly using tumour tissues [9, 10]. It is relatively difficult to obtain metastatic tissues from advanced patients, and the gene status of a single tumour site cannot reveal the genetic panorama of tumours throughout the body. Importantly, genotyping circulating tumour DNA (ctDNA) is useful for detecting the overall view of genomic alterations, especially in patients with heterogeneous metastatic sites [11–14].

**Fig. 1:**
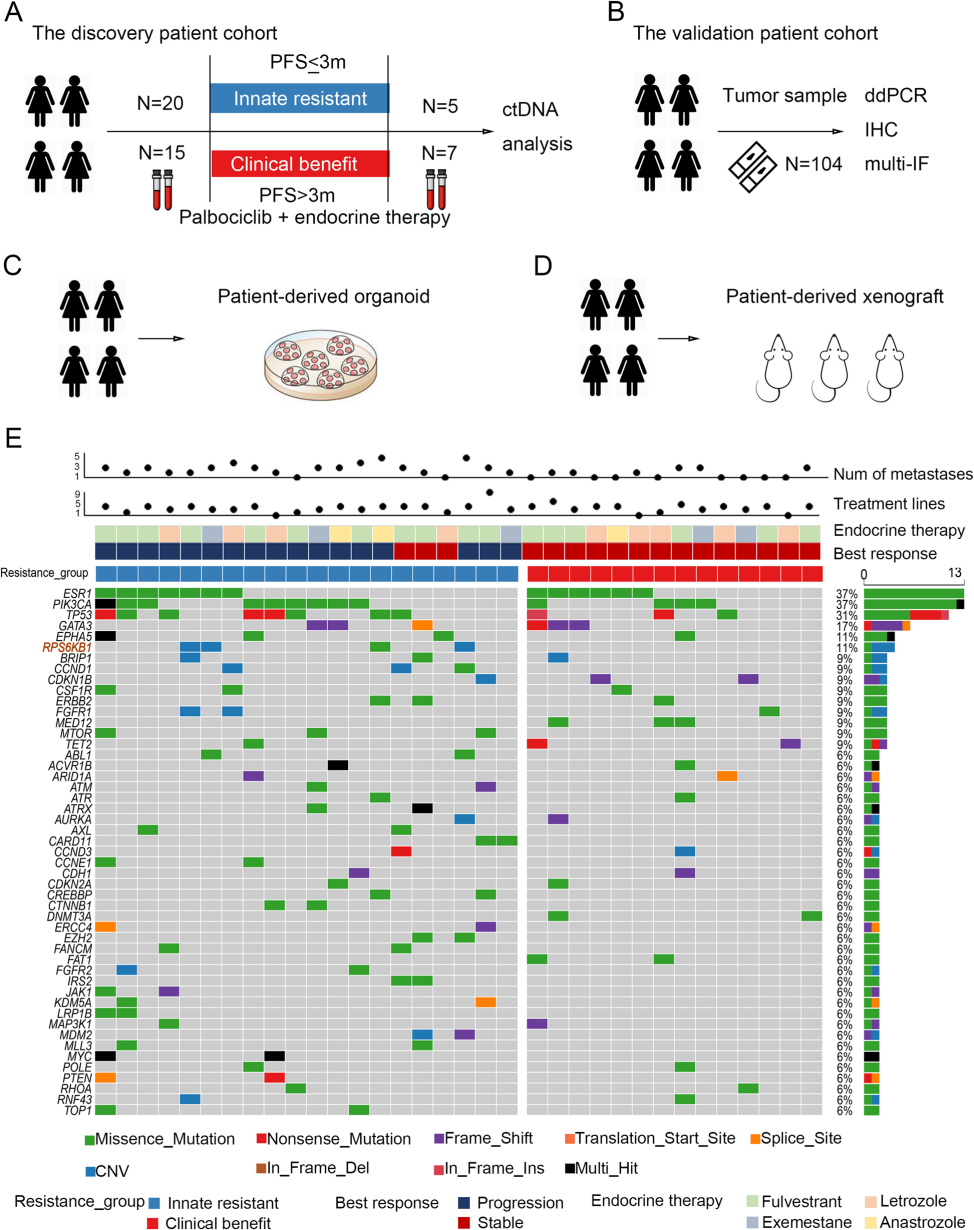
The key genes related to the innate resistance of CDK4/6 inhibitors were explored by analysis of ctDNA derived from patients with breast cancer. **A**-**D** Schematic overview of the study design and analytical workflow. PFS: progression-free survival. ctDNA: circulating tumour DNA. ddPCR: droplet digital PCR. IHC: immunohistochemistry. Multi-IF: multiplex immunofluorescence. **E** The landscape of high-frequency altered genes in plasma from the discovery patient cohort. The plot shows patients with innate resistance or clinical benefit following palbociclib treatment, with individual information about numbers of metastases, treatment lines, and endocrine therapy drugs used in combination with palbociclib. S6K1 (i.e. RPS6KB1) amplification was observed in three patients with innate resistance to palbociclib but not in those with clinical benefit

Therefore, we performed ctDNA testing in a real-world cohort of patients with ER^+^HER2^−^ MBC (Fig. 1A) to search for biomarkers that could predict innate resistance to CDK4/6 inhibitors, and validated the candidate biomarker in another independent patient cohort (Fig. 1B). Using cellular experiments, we investigated the molecular mechanisms of the key genetic abnormality responsible for conferring resistance to CDK4/6 inhibitors. Furthermore, based on the results of patient-derived organoid (PDO) and patient-derived xenografts (PDX) studies (Fig. 1C and D), we proposed a potential therapeutic strategy to overcome innate resistance to CDK4/6 inhibitors.

## Methods

### Patients and sample collection

A total of 186 patients visited Cancer Hospital, Chinese Academy of Medical Sciences and were prescribed palbociclib between May 2016 and November 2019. Among them, 36 patients agreed to draw blood for ctDNA analysis on the first day of treatment, and constituted the discovery cohort of this study (NCC Protocol #1787). The independent validation cohort consisted of ER^+^ breast cancer patients whose tissue sample was available in the Institutional Tumour Bank. A total of 104 patients were included in this study, 37 of whom had received palbociclib treatment. Blood and tissue samples were all obtained with appropriate written informed consent. Clinical data were collected and reviewed in electronic medical charts retrieved from the database of the China National Cancer Center. Computed tomography (CT) scans were obtained using standard procedures in the Cancer Hospital, Chinese Academy of Medical Sciences as part of the routine clinical care of patients. RECIST 1.1 measurements were performed by a physician formally trained in tumour metrics. This study was approved by the Ethics Committee of Cancer Institute and Hospital, Chinese Academy of Medical Sciences (12-123/657). All study participants provided informed consent.

PFS was defined as the period from the initiation of palbociclib administration to the date of disease progression, which was determined by the physician based on radiological information. Clinical benefit was defined as PFS > 3 months and innate resistance was defined as PFS ≤ 3 months following initiation of palbociclib administration. A PFS of 3 months was chosen as the cut-off value because most patients had been heavily treated; moreover, this cut-off period was based on a previous study [15]. The baseline characteristics of patients were categorised and compared using Fisher’s exact tests.

### Sequencing and bioinformatics analysis

Plasma DNA from 36 patients with MBC was analysed using a customised panel of 1021 cancer-related genes [16]. Genes targeted by the panel are listed in Supplementary Table S1. Samples were prepared following the standard laboratory procedures and the manufacturer’s protocols. DNA extraction, library preparation, hybrid capture, and sequencing were performed as previously described [17]. Somatic single nucleotide variants were analysed using the GATK toolkit version 3.4-46-gbc02625 and subjected to filtering. Somatic copy number variants were analysed using the CONTRA 2.0.8 software, and the matched peripheral blood cell samples were used as matched controls.

Whole exome sequencing data of patients with MBC from the INSERM cohort [18] and the Metastatic Breast Cancer Project (https://www.mbcproject.org/, a project of Count Me In (https://joincountmein.org/)) [19], and patients with primary breast invasive carcinoma from The Cancer Genome Atlas (TCGA, Firehose Legacy) were used to analyse the prevalence of *S6K1* gene amplification in breast cancer using cBioPortal (http://www.cbioportal.org/). For pan-cancer analysis, the gene alteration data involved 10,967 tumour samples from 32 TCGA PanCancer Atlas Studies was obtained from cBioPortal. The RNA-seq data in 17 cancer types from TCGA was downloaded from the Human Protein Atlas.

For differentially expressed gene (DEG) analysis, we retrieved expression levels of genes in terms of count values from the BRCA cohort through the PanCancer Atlas consortium publication page. We applied R (EdgeR) package [20] to calculate the relative fold changes of high S6K1 expression subtype versus low S6K1 expression. The Gene Set Enrichment Analysis (GSEA) method was performed using the cluster Profiler package to identify hub genes and significant common pathways [21]. For statistical analysis, *t*-test, Fisher’s exact test and Spearman test were performed. A two-sided *P* value < 0.05 was considered statistically significant. The R 3.6.1 package was used for all the analyses.

For RNA sequencing, total RNA was extracted from MCF-7 cells transfected with S6K1 siRNA pool or non-sense control siRNA by using TRIzol Reagent (Invitrogen). Next, 2 μg total RNA was used for RNA sequencing library preparation using KCTM Stranded mRNA Library Prep Kit (Wuhan Seqhealth Co., Ltd. China) following the manufacturer’s instruction. PCR products from 200 to 500 bps were enriched, quantified and finally sequenced on Novaseq 6000 sequencer (Illumina) with PE150 model.

### Immunohistochemical staining

FFPE specimens from patients with breast cancer were collected at Cancer Hospital, Chinese Academy of Medical Sciences. Of a total of 104 samples, 61 were from metastases and the other 43 were from primary tumors. Incubation with polyclonal antibodies against S6K1 (diluted at 1:1000, Servicebio, Cat# GB111181) was performed at 4 °C for 18 h. They were incubated with the secondary antibody (diluted at 1:200, Servicebio, Cat# GB23303) for 50 min at room temperature. Quality assessment was performed on each batch of slides by including a negative control in which the primary antibody was replaced by 10% normal goat serum to preclude non-specific signals. Staining was assessed by pathologists who were blinded to the sample origins and the patient outcomes. Each specimen was assigned a score according to the intensity of the cytoplasmic staining (no staining = 0; weak staining = 1, moderate staining = 2 and strong staining = 3) and the extent of stained cells (0–5% = 0, 6–25% = 1, 26–50% = 2, 51–75% = 3 and 76–100% = 4). The final immunoreactivity positive score was determined by multiplying the intensity score by the score for the extent of stained cells, generating a score that ranged from 0 (the minimum score) to 12 (the maximum score). All patients were divided into high (positive score ≥ 6) and low (positive score < 6) expression subgroups. Log-rank test were used for the comparison of survival curves.

### Droplet digital PCR (ddPCR)

A customized multiplex digital PCR assay was employed to assess copy number variation (CNV) of *S6K1* gene in FFPE samples on the OS-300 digital PCR system (Dawei Biotech, China) using OsciDrop technique [22]. Paraffin from FFPE slides was removed with deparaffinization solution. Genomic DNA was extracted using the GeneRead DNA FFPE Kit (Qiagen N. V., Germany). DNA was eluted in 60 μL Buffer ATE (Qiagen), quantified with NanoDrop One spectrophotometer (Thermo Fisher Scientific, USA), and diluted using nuclease-free water to 10 ng/μL for use. The ddPCR reaction mixture (25 μL) was prepared in 0.2 mL tubes, consisting of 12.5 μL 2X ddPCR Multiplex Supermix (Dawei Biotech), 2.5 μL primers and probes for *S6K1* CNV detection (Dawei Biotech), 0.65 μL DNA Polymerase, 2.5 μL DNA template, and 6.85 μL nuclease-free water. The PCR program included 5 min activation at 95 °C, followed by 45 cycles of 20 s denaturation at 94 °C and 60 s annealing at 58 °C, and finally held at 25 °C.

To set a cut-off for definition of positive *S6K1* amplification, an averaged copy-number ratio of the *S6K1* gene relative to the reference gene, *CEP17*, from 26 breast cancer tissues with low S6K1 IHC expression (positive score < 6) was determined (Supplementary Table S2). A cut-off for a positive sample was set at 1 SD above the mean (threshold, 3.546).

### Multiplex immunofluorescence (multi-IF)

To evaluate the expression level of S6K1 as well as cell cycle-related proteins in tumours, FFPE slides from ER^+^HER2^−^ breast cancer patients among the validation cohort of patients prescribed with palbociclib were subjected to multi-IF and multispectral imaging using antibodies targeting cyclin E1 (Abcam, Cat# ab135380), cyclin D1(CST, Cat# 2978 T), phospho-Rb (CST, Cat# 8516 T), S6K1 (CST, Cat# 2708 T), CDK4 (Proteintech, Cat#11026-1-AP), and CDK6 (Abcam, Cat# ab124821). After each primary antibody was sequentially applied, the slides were incubated with secondary antibodies, followed by tyramide signal amplification (TSA). Nuclei were stained with DAPI after all the human antigens had been labelled. To obtain multispectral images, the stained slides were scanned using the Mantra System (PerkinElmer), which captured the fluorescence excitation spectrum at 20-nm wavelength intervals (420–720 nm) within the same exposure time. Multiple scans were combined to build a single stack image. The spectrum of auto-fluorescence of the tissues and each fluorescein was extracted from images of unstained and single-stained sections. A spectral library required for multispectral unmixing was established by InForm image analysis software (Version 2.4, PerkinElmer). The reconstructed images were then obtained with the auto-fluorescence removed. The Spearman correlation analysis was used to analyse the correlations between the expression of S6K1 and cell-cycle relevant markers. The Mann-Whitney test was performed for comparison of the proportions of specific cell subsets.

### Cell culture and drugs

T47D and MCF-7 cell lines were obtained from American Type Culture Collection (ATCC) and were maintained in DMEM (Cell Technologies) with 10% foetal bovine serum at 37 °C in a humidified atmosphere containing 5% CO_2_. These two cell lines have been validated by the short tandem repeat (STR) method. Inhibitors used in the treatment of cells, including palbociclib (HYA0065) and rapamycin (HY-10219), were purchased from MedChemExpress (MCE).

### Cell proliferation and drug sensitivity assays

Cell proliferation was examined using the xCELLigence Real-Time Cell Analyser (RTCA)-Multiple Plates (MP) system (Acea Biosciences/Roche Applied Science), which can measure the growth status of cells in real time. MCF-7 (3 × 10^3^ cells/well) or T47D (1 × 10^4^ cells/well) cells were plated in 96-well electronic microplates (E-Plate 96, Roche Applied Science). Then, the E-Plate 96 was placed in the RTCA-MP device and cultured at 37 °C with 5% CO_2_. Every 15 min, the cell index was detected automatically according to the change of electrical impedance. For the drug sensitivity assay, the cells were first cultured in complete medium for the indicated period, and the culture medium was subsequently replaced with different concentrations or types of additive drugs.

CCK-8 and clonogenic assays were also used to evaluate the drug sensitivity. For CCK-8 assay, MCF-7 or T47D cells were plated in 96-well plates. After 12 h, the cells were treated with increasing doses of palbociclib for 5 days. Then, CCK-8 (NCM Biotech) assay was performed according to the manufacturer’s instructions. For clonogenic assay, MCF-7 or T47D cells were plated in 12-well plates and treated with palbociclib alone or in combination with rapamycin. After 10 days, the cells were fixed and stained with crystal violet.

### Western blotting and antibodies

Total proteins extracted from breast cancer cells were resolved by SDS-PAGE (10%) and transferred to PVDF membranes. After blocking with 5% non-fat milk, the membranes were incubated with the indicated primary antibodies overnight at 4 °C and the secondary antibodies for 1 h at room temperature. The primary antibodies used in this study were as follows: S6K1 (Proteintech, Cat# 14485-1-AP), cyclin D1 (Proteintech, Cat# 60186-1-1 g), cyclin E1 (Abcam, Cat# ab33911), phospho-Rb (Abcam, Cat# ab109399), CDK2 (Proteintech, Cat# 10122-1-AP), CDK4 (Santa Cruz Biotechnology, Cat# sc-260), CDK6 (CST, Cat# 3136S), c-Myc (CST, Cat# 5605S), β-Actin (CST, Cat# 3700S), and GAPDH (Proteintech, Cat# 60004-1-Ig).

### Small interfering RNA transfection and lentivirus infection

Cells were transfected with siRNAs targeting S6K1 (RiboBio #stB0004595A: GATGAGAAGTGGCCACAAT; #stB0004595B: GGACGCTGGAGAAGTTCAA; #stB0004595C: GAGTTGGACCATATGAACT) or non-sense control siRNA using Lipofectamine 2000 (Invitrogen) according to the manufacturer’s instructions.

To establish a stable exogenous S6K1-expressed T47D cell line, HEK293T cells were used for S6K1-lentivirus packaging. Then, the lentiviral supernatant was collected and added to the T47D cell culture medium in a 1:1 ratio, followed by screening with puromycin.

### Reverse transcription-quantitative polymerase chain reaction (RT-qPCR)

Total RNA of breast cancer cells was isolated with TRIzol reagent (Invitrogen) and was reverse-transcribed to cDNA using M-MLV reverse transcriptase (Promega). Quantitative PCR was performed using the BrightGreen 2X qPCR MasterMix (abm) on the CFX96 Real-Time System (Bio-Rad). GAPDH was served as the internal control. The primers used in this study are the follows: S6K1, forward 5′-GCCTCCCTACCTCACACAAG-3′, reverse 5′-CCACCTTTCGAGCCAGAAGT-3′; c-Myc, forward 5′-GGCTCCTGGCAAAAGGTCA-3′, reverse 5′-CTGCGTAGTTGTGCTGATGT-3′; cyclin E1, forward 5′-GCCAGCCTTGGGACAATAATG-3′, reverse 5′-CTTGCACGTTGAGTTTGGGT-3′; GAPDH, forward 5′-GCTGAGAACGGGAAGCTTGT-3′, reverse, 5′-GCCAGGGGTGCTAAGCAGTT-3′.

### Cell cycle analysis

The cells were collected, washed with PBS, and fixed in 70% ethanol overnight at − 20 °C. Next, the cells were washed with PBS and stained with 500 μL of PI/RNase Staining Buffer (BD Biosciences) for 15 min at 37 °C, followed by analysis using a flow cytometer (BD Biosciences).

### Chromatin immunoprecipitation (ChIP)

ChIP assay was carried out by using Pierce Magnetic ChIP Kit (Thermo Fisher Scientific) following the manufacturer’s instructions. The antibody against c-Myc (CST, Cat# 9402) was incubated with digested chromatin of MCF-7 cells followed by addition of magnetic beads. After purifying the coprecipitated DNA, qPCR was used to detect the promoter of CCNE1 with primers as follows:

CCNE1-promoter-1 (P1), forward 5′-AAGGACTTAGCCCAGTGCTG-3′, reverse 5′-CATCCTGTGCCCGTTAGGAAT-3′; CCNE1-promoter-2 (P2), forward 5′-GTCAGAAAGGTCTTCAGAGAGCC-3′, reverse 5′-TGTCCATTCATCCGTCAGTGC-3′; CCNE1-promoter-3 (P3), forward 5′-CCACACATCCCCTTGGCTCA-3′, reverse 5′-GCGCGGGTGGAATGTAAACA-3′.

### Luciferase reporter assay

The pGL3-CCNE1-promoter vector was obtained by inserting the promoter region of CCNE1 from − 1 to − 2000 bp into pGL3-basic vector. MCF-7 cells were co-transfected with pGL3-CCNE1-promoter vector and pRL-TK vector, or pGL3-basic control vector and pRL-TK vector, together with siRNAs targeting S6K1 or control siRNAs by using Lipofectamine 2000 (Invitrogen). Then, the transfected cells were lysed to measure the firefly and Renilla luciferase activities with a Dual Luciferase Reporter Assay System (Promega). The activity of Renilla luciferase was used as control for firefly luciferase activity.

### PDO study

Breast cancer organoids derived from patients were established using previously described methods [23]. Breast cancer tissues were obtained from consenting patients of a clinical study (NCT03544047). Upon receipt, the tissues were minced with scissors and digested in an enzyme mixture of collagenase (Sigma-Aldrich, 1 mg/ml) and dispase (Sigma-Aldrich, 1 mg/ml) for 1–2 h at 37 °C. The separated breast cancer cells were mixed with ice-cold growth factor-reduced Matrigel (Corning), seeded in culture plates and incubated at 37 °C for 30 min. The surface of the solidified mixture of cell suspension/Matrigel was sealed with complete breast cancer organoid medium, which comprised advanced DMEM/F12 supplemented with several additives, as described by Sachs et al. [23]. Breast cancer organoids were digested bi-weekly using TrypLE (Gibco). The dissociated organoids were then subjected to passage or compound evaluation.

Breast cancer organoids were plated in 96-well plates with Matrigel for 2 days, followed by treatment with serially diluted palbociclib alone or in combination with mTOR inhibitor everolimus for 96 h. Cell viability was determined using the CellTiter-Glo® assay (Promega). Dose–effect curves were generated using GraphPad Prism software (version 8.0). The synergistic effect of palbociclib combined with mTOR inhibitor was assessed using a dose–response matrix [24]. Bliss synergy scores were calculated and plotted using the SynergyFinder package in R [24, 25].

### PDX study

A breast cancer patient tumour specimen with *S6K1* gene amplification was obtained from the commercial vendor Crown Bioscience in China. Approximately 20-30 mg of tissue fragments were implanted subcutaneously into 9 NOD.SCID mice. Mice were maintained and handled in accordance with the animal care and use committee–approved animal protocols. Mice bearing tumours measured ≥150mm^3^ were randomized to treatment with (1) vehicle (control), (2) palbociclib (50 mg/kg, daily, p.o.), or (3) palbociclib plus rapamycin (6 mg/kg, daily, i.p.). Animal weights and tumour diameters (using callipers) were measured twice weekly. Four weeks later, all mice were executed and tumours were made into FFPE slides for immunohistochemical staining, using antibodies against cyclin E1 (Abcam, Cat# ab135380), cyclin D1(CST, Cat# 2978 T), phospho-Rb (CST, Cat# 8516 T), S6K1 (Servicebio, Cat# GB111181), and Ki67 (Servicebio, Cat# GB13030-M-2).

### Statistical analysis

Statistical analysis was performed as described in experiment. Data are represented as the mean ± SD unless otherwise stated in the figure legend. *P* value was calculated by two-sided Student’s *t*-test between two groups, unless otherwise specified. ANOVA (no missing value) or Mixed-effects model (if there are missing values) was used for the comparison among multiple groups. *P* < 0.05 was considered statistically significant (GraphPad Prism; ****, *P* < 0.0001; ***, *P* < 0.001; **, *P* < 0.01; *, *P* < 0.05).

## Results

### Patients and clinical characteristics

To evaluate somatic alterations associated with clinical resistance of breast cancer to CDK4/6 inhibitor, 36 patients with ER^+^HER2^−^ MBC received palbociclib and endocrine therapy were included in this study as the discovery cohort (Fig. 1A). One patient discontinued palbociclib due to severe myelosuppression after 4 weeks of treatment, whose resistance to palbociclib was not determined. Therefore, resistance-related ctDNA analysis included a total of 35 patients subsequently.

The clinical and pathological characteristics of patients in the discovery cohort are summarised in Supplementary Table S3. Of the 36 patients, 18 (50.0%) received palbociclib plus fulvestrant, 10 (27.8%) received palbociclib plus letrozole, 5 (13.9%) received palbociclib plus exemestane regimen, and 3 (8.3%) received the combination of palbociclib and anastrozole. These 36 patients had previously received a median of 3 (range 0–8) lines of systemic treatment for metastatic breast cancer before palbociclib therapy. Only 4 (11.1%) patients received CDK4/6 inhibitor-based therapy as first-line treatment for their metastatic disease. Nineteen patients (52.8%) received at least 3 lines of systemic treatment previously. Thirty-two patients experienced tumour progression following palbociclib therapy, while the other 3 patients were still being treated at the time of analysis. Fifteen patients showed clinical benefit from CDK4/6 inhibitor and experienced disease progression for more than 3 months after the initiation of treatment. Meanwhile, 20 patients developed innate resistance (PFS ≤ 3 months) to palbociclib. There were no significant differences in the clinical and pathological characteristics of patients between the ‘clinical benefit’ (*n* = 15) and ‘innate resistance’ (*n* = 20) groups (Supplementary Table S3).

### ctDNA profiling reveals various mechanisms of palbociclib resistance

A total of 47 plasma samples was analysed for ctDNA profiling as our discovery cohort, using a panel comprising 1021 cancer-related genes: 35 samples were collected before palbociclib treatment, and 12 samples were collected after disease progression (Fig. 1A). The detected molecular alterations and their mutant allele fractions in ctDNA are presented in Supplementary Table S4.

We firstly investigated the potential biomarkers of palbociclib resistance based on the detected molecular alterations of ctDNA profiled from plasma that was collected before initiation of palbociclib. Out of the 35 baseline samples, 34 (97.1%) had at least one somatically genomic alteration in cancer-related genes, with a mean of 8 alterations (range 0–24). The landscape of high-frequency altered genes (> 5%) in patients of the ‘innate resistance’ and ‘clinical benefit’ groups is presented in Fig. 1E. Consistent with previous reports [12], *ESR1*, *PIK3CA,* and *TP53* were the most frequently mutated genes in MBC. *ESR1* mutations were detected in 13 patients (13/35, 37.1%); the most common mutation was D538G (6/35), followed by Y537S (4/35) and L536H (3/35). *PIK3CA* mutations were detected in 13 patients (13/35, 37.1%); the most common mutation was H1047R (8/35), followed by E545K (3/35). *TP53* mutations were detected in 11 patients (11/35, 31.4%); the most common mutation was R213* (2/35). In the 20 patients with innate resistance to palbociclib, mutations were also mostly detected in *PIK3CA* (9/20, 45.0%), *TP53* (8/20, 40.0%), and *ESR1* (7/20, 35.0%). The mutated loci of these genes were similar in patients with or without innate resistance to palbociclib (Supplementary Fig. S1A).

Diverse PI3K-pathway activating events were observed in 19 patients, including *PIK*, *S6K1*, *MTOR*, *PTEN*, *AKT*, and *TSC1* (Supplementary Fig. S1B). The frequency of PI3K pathway alterations in the innate resistance group (14/20, 70.0%) was significantly higher than that in the clinical benefit group (5/15, 33.3%; *P* = 0.044), suggesting that altered PI3K signalling is a potential mechanism for innate resistance to palbociclib therapy. Notably, genetic alterations in *S6K1* (4/20, 20.0%), *CCND1* (3/20, 15.0%), and *MTOR* (3/20, 15.0%) were enriched in patients with innate resistance but not detected in those with clinical benefit from palbociclib (Fig. 1E; Supplementary Fig. S1C), thereby suggesting that they might be contributing to innate resistance to palbociclib.

ctDNA samples from 12 patients were tested both at the baseline and after disease progression. Significant differences in genomic alternations were found between the samples at baseline and after disease progression (Supplementary Fig. S1D). Of the 7 patients who had benefited from palbociclib treatment, 3 patients demonstrated new mutations in genes after disease progression: one in *TSC2*, one in *PTEN*, while the other one had de novo mutations in *ESR1*, *RB1,* and *TP53*. These results suggest that these genes might be contributing to the acquired resistance to palbociclib.

### *S6K1* amplification contributes to palbociclib resistance in patients with breast cancer

In the ctDNA analysis of our discovery cohort, *S6K1* amplification (threshold = 2.5 copy number) was detected in 3 patients (3/20, 15.0%) with innate resistance to palbociclib at the baseline, but not in those with clinical benefit (0/15, Fig. 1E). During the analysis of interactions between the high-frequency altered genes, *S6K1* appeared to be mutually exclusive to *PIK3CA* in patients with innate resistance to palbociclib (Supplementary Fig. S1E). The three patients with *S6K1* amplification at baseline were all pre-treated with more than two lines of endocrine therapy for their metastatic disease. Meanwhile, *S6K1* amplification was not found in the plasma samples collected after palbociclib treatment, including those with acquired resistance to palbociclib. Therefore, *S6K1* may be a novel candidate to understand the innate resistance mechanism.

**Fig. 2:**
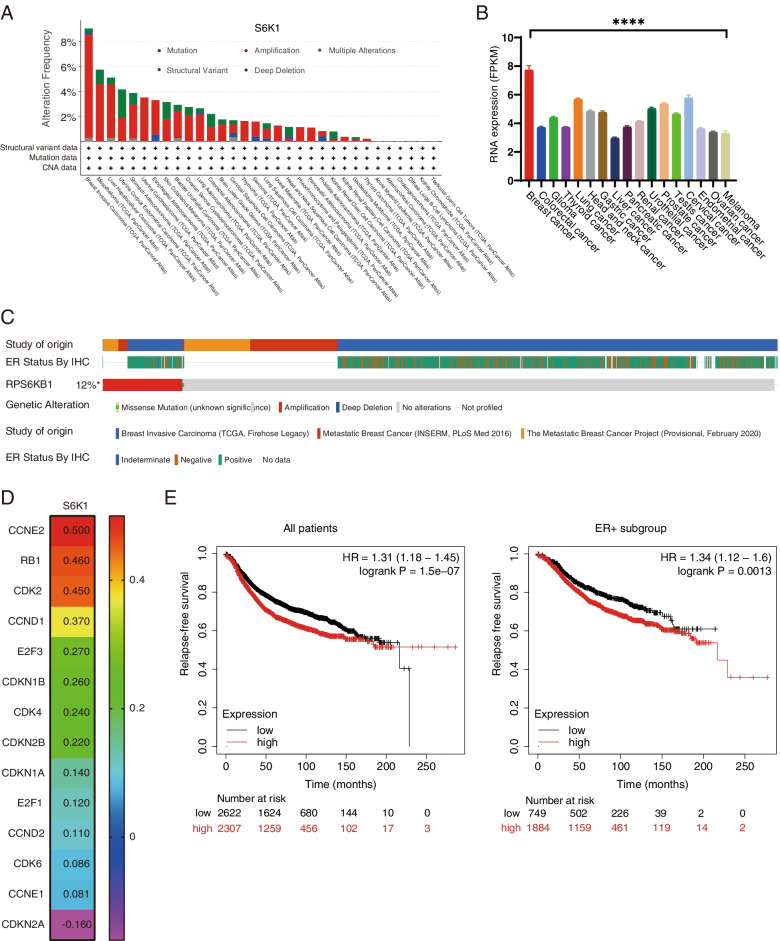
*S6K1* amplification correlates with poor outcome in patients with breast cancer from public database. **A** The incidence of *S6K1* genetic alteration in different cancer types from 10,967 samples, obtained by summarizing the data of TCGA PanCancer Atlas Studies on cBioPortal. **B** The mRNA level of *S6K1* gene in TCGA patients with various cancers are reported as median FPKM (Fragment Per Kilobase of exon per Million reads), downloaded from the Human Protein Atlas. Data are represented as the mean ± SEM. *P* value was calculated by Kruskal-Wallis test. ****, *P* < 0.0001. **C** The *S6K1* locus was substantially amplified in breast cancer samples from oestrogen receptor (ER) positive subtypes based on cBioPortal. **D** The correlations between the mRNA levels of *S6K1* and cell cycle-related genes in BRCA cohort, using data from the GEPIA. **E** Kaplan-Meier Plotter showing the relapse free survival of patients with breast cancer according to S6K1 mRNA levels

*S6K1* gene is an important component of the PI3K signalling pathway, which is located on chromosome 17q23. Using the sequencing data from The Cancer Genome Atlas (TCGA), we found that high-levels of *S6K1* gene amplification and RNA expression are mainly limited to breast cancer (*P* < 0.0001, Fig. 2A and B). The meta-analysis of the data using cBioPortal showed that *S6K1* amplification is observed in approximate 12% of patients with breast cancer, mainly in the estrogenic receptor positive subtype (Fig. 2C). The incidence of *S6K1* amplification is 14% in those with MBC and 11% in patients with primary disease (Supplementary Fig. S2A). Amplification of *S6K1* gene led to a significant increase in its mRNA expression, which was in turn correlated with the high expression of S6K1 protein in breast cancers (Supplementary Fig. S2B). Consistent with the results from our discovery cohort, analysis of breast cancer data from the TCGA PanCancer Atlas showed that the S6K1-PIK3CA pair had a tendency toward mutual exclusivity (*P* = 0.030, Log2 odds ratio = − 0.679, Supplementary Table S5). A total of 210 mRNAs were upregulated and 406 mRNAs were downregulated by at least two-fold in the S6K1-overexpressed breast cancer samples from TCGA database. In the *S6K1* gene high expression group, several pathways including the cell cycle pathway were significantly enriched (Supplementary Fig. S2C). Based on the gene expression levels of the BRCA cohort from the TCGA database by using the GEPIA, we observed that *S6K1* gene is closely related to the RNA expression of the cell cycle pathway genes, especially the CCNE2 (*P* < 0.001, *r* = 0.50), RB1 (*P* < 0.001, *r* = 0.46), CDK2 (*P* < 0.001, *r* = 0.45), CCND1 (*P* < 0.001, *r* = 0.37), and CDK4 (*P* < 0.001, *r* = 0.24, Fig. 2D; Supplementary Fig. S2D). Kaplan-Meier Plotter [26] showed that breast cancer patients with high *S6K1* expression had significantly worse relapse-free survival (hazard ratio = 1.31, *P* < 0.0001), especially in the ER^+^ subgroup (hazard ratio = 1.34, *P* = 0.0013, Fig. 2E).

**Fig. 3:**
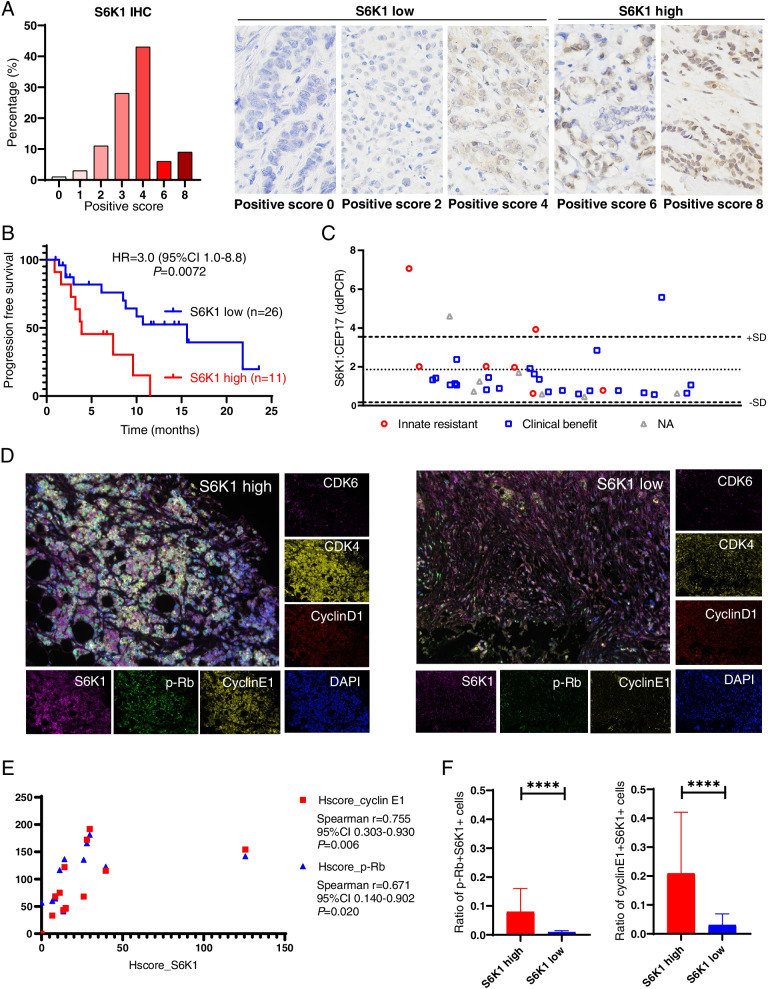
Elevated expression of S6K1 is related to palbociclib resistance in the validation patient cohort. **A** The expression of S6K1 in patients with breast cancer was detected by immunohistochemistry (IHC). High S6K1 expression (positive score 6-8) was observed in 15/104 (14%) of patients. **B** Kaplan–Meier curves for PFS in the validation patient cohort who had received palbociclib treatment according to S6K1 expression. *P* value was calculated by log-rank test. **C** Copy number variation profiles for the *S6K1* loci using droplet digital PCR (ddPCR). **D** Representative images showing multiplex immunofluorescence (multi-IF) stanning of tissue samples from patients with high or low S6K1 expression. **E** The expression of S6K1 had a high correlation with cyclin E1 and p-Rb based on the results of multi-IF in the patient with high S6K1 expression. **F** The proportion of p-Rb + S6K1+ cells and cyclin E1 + S6K1+ cells in patients with high or low S6K1 expression. Data are represented as the mean ± SD. *P* value was calculated by Mann-Whitney test. ****, *P* < 0.0001

We then investigated the relationship between S6K1 and palbociclib resistance using an independent validation cohort of 104 patients with ER^+^MBC. First, we evaluated the expression level of S6K1 using immunohistochemistry (IHC). Figure 3A shows that S6K1 was overexpressed in 15/104 (14%) patients with an IHC positive score of 6-8. Thirty-seven out of these 104 patients had received palbociclib treatment (Supplementary Table S6), and 29.7% (11/37) of them showed highly expressed S6K1. These patients with high-expressed S6K1 had significantly worse PFS than those with low S6K1 expression (median PFS 3.9 vs 15.6 months, hazard ratio = 3.0, 95% CI 1.0-8.8, *P* = 0.0072, Fig. 3B). Using the ddPCR technique, we found that 4 out of these 37 patients had *S6K1* amplification (Fig. 3C). The incidence of *S6K1* amplification was 28.6% (2/7) in patients who had innate resistance to palbociclib and only 4.3% (1/23) in those with clinical benefit after palbociclib treatment. Next, we performed multiplex immunofluorescence (multi-IF) staining of multiple cell cycle-related markers in patients with high or low S6K1 expression. We found that the expression of S6K1 had a high correlation with the levels of cyclin E1 (*r* = 0.755. *P* = 0.006) and phosphorylated Rb (p-Rb) (*r* = 0.671, *P* = 0.020, Fig. 3D and E). In patients with highly expressed S6K1, the proportion of p-Rb + S6K1+ cells (median 5.1% vs 0.8%, *P* < 0.0001), as well as the proportion of cyclinE1+ S6K1+ cells (median 12.2% vs 1.6%, *P* < 0.0001), were significantly higher than that of patients with low S6K1 expression (Fig. 3F).

Collectively, these data show that *S6K1* amplification is prominent in patients with breast cancer. Elevated expression of S6K1 might play an important role in the resistance mechanisms of palbociclib via the cell cycle pathway.

### S6K1 overexpression promotes cell proliferation and the resistance to CDK4/6 inhibitors

**Fig. 4:**
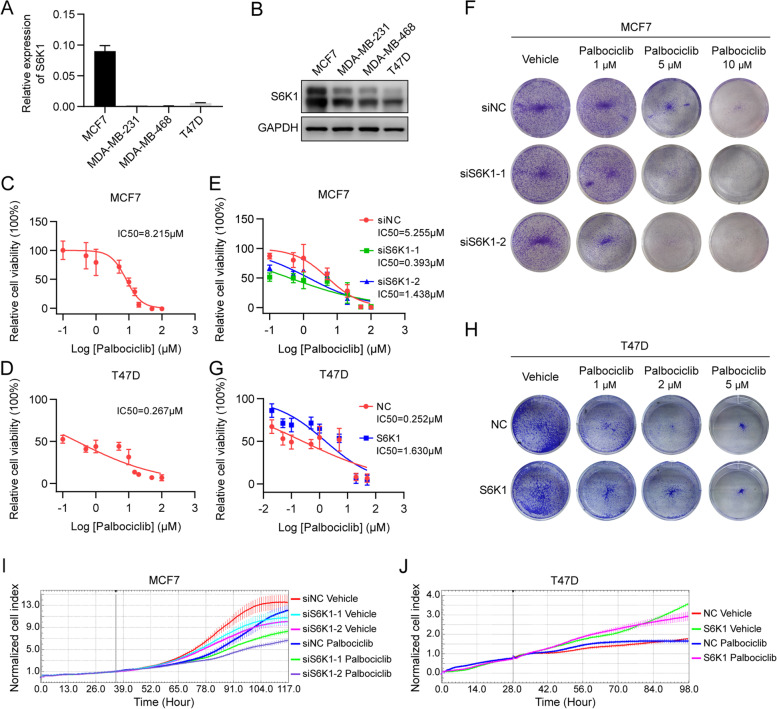
S6K1 confers resistance to palbociclib in breast cancer cell lines. **A**, **B** The expression of S6K1 was measured in four breast cancer cell lines, including MCF-7, MDA-MB-231, MDA-MB-468, and T47D, using RT-qPCR (**A**) and western blotting (**B**), respectively. **C**, **D** MCF-7 (C) or T47D (D) cells were treated with increasing doses of palbociclib, as indicated, for 5 days. The cell viability was then detected using CCK-8 assay. **E**, **F** MCF-7 cells were transfected with two specific S6K1 siRNAs (siS6K1-1/− 2) or non-sense control siRNA (siNC), followed by CCK-8 assay (**E**) or clonogenic assay (**F**) upon treatment with increasing doses of palbociclib as indicated. **G**, **H** Exogenous S6K1-expressed T47D cells or control cells were subjected to CCK-8 assay (**G**) or clonogenic assay (**H**) with the indicated doses of palbociclib. **I**, **J** MCF-7 cells transfected with two specific S6K1 siRNAs or non-sense control siRNA (**I**) as well as exogenous S6K1-expressed T47D cells or control cells (**J**) were treated with or without palbociclib (10 μM for MCF-7 cells, 1 μM for T47D cells). The xCELLigence system was used for examining proliferation of these cells

We next examined S6K1 expression in T47D, MCF-7, MDA-MB-468, and MDA-MB-231 breast cancer cells. MCF-7 cells showed the highest S6K1 expression both at the mRNA and protein levels (Fig. 4A and B). Consistently, according to the Cancer Cell Line Encyclopedia (CCLE) database [27], S6K1 mRNA expression in MCF-7 cells was remarkably higher than that in most other breast cancer cell lines; the reason for this high expression in MCF-7 cells is high level of *S6K1* DNA copy number (Supplementary Fig. S3A). Thus, we selected MCF-7 and T47D cells, both of which were ER^+^HER2^−^ breast cancer cells, to further explore whether *S6K1* amplification confers resistance to CDK4/6 inhibitors in vitro. As expected, MCF-7 cells were less sensitive to palbociclib than T47D cells (Fig. 4C and D). Furthermore, small interfering RNA (siRNA)-mediated knockdown of S6K1 in MCF-7 cells (Supplementary Fig. S3B and C) restored the sensitivity to palbociclib (Fig. 4E and F). Conversely, lentivirus-infected, exogenous S6K1-expressed T47D cells showed reduced sensitivity to palbociclib compared to the control cells (Fig. 4G and H, Supplementary Fig. S3D and E). These results suggest that S6K1 overexpression, caused by gene amplification, promotes resistance to palbociclib in ER^+^HER2^−^ breast cancer cells.

Moreover, we examined the function of S6K1 in cell proliferation by using the xCELLigence system. Knockdown of S6K1 in MCF-7 cells inhibited their proliferation with or without palbociclib treatment (Fig. 4I). Furthermore, exogenous expression of S6K1 promoted T47D cell proliferation, even with palbociclib treatment (Fig. 4J).

### Activation of c-Myc/cyclin E1 pathway may mediate S6K1-induced drug resistance

**Fig. 5:**
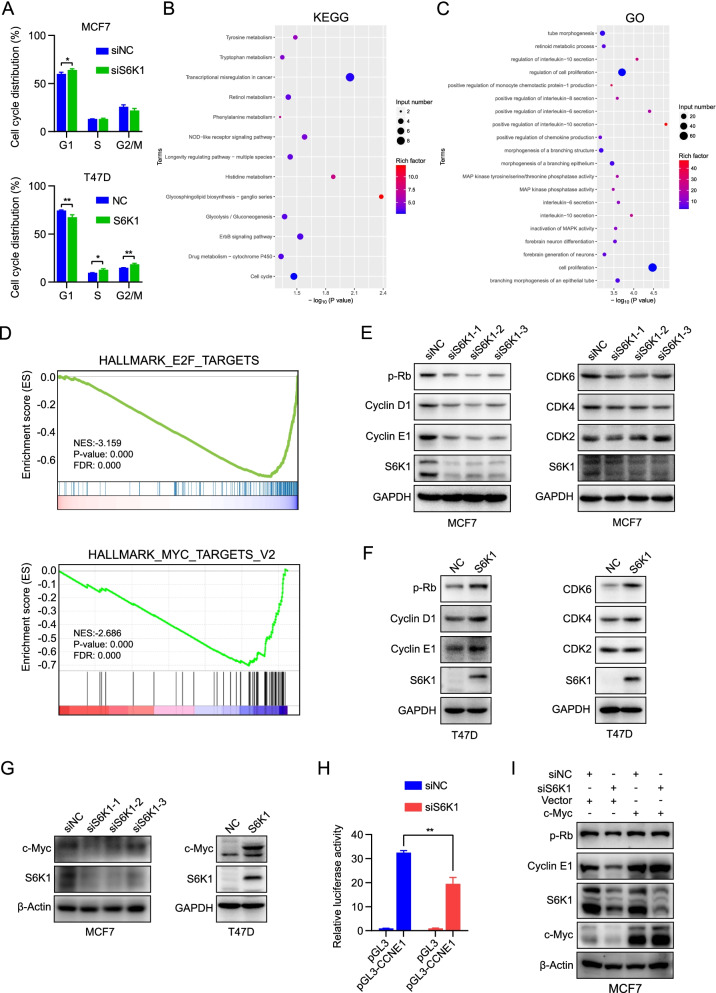
S6K1 promotes cell proliferation and palbociclib resistance via cell cycle progression. **A** Cell-cycle distribution was measured using PI staining followed by flow cytometry for MCF-7 cells transfected with S6K1 siRNA pool or non-sense control siRNA as well as exogenous S6K1-expressed T47D cells or control cells, respectively. *P* value was calculated by Student’s *t*-test. *, *P* < 0.05, **, *P* < 0.01. **B**, **C** KEGG pathway enrichment analysis (**B**) and GO functional analysis (**C**) of RNA-seq data obtained from S6K1-depleted MCF-7 cells. **D** GSEA showing an enrichment of E2F-targets and Myc-targets signatures in S6K1-depleted MCF-7 cells. **E**-**G** MCF-7 cells transfected with the indicated siRNAs as well as exogenous S6K1-expressed T47D cells or control cells with 70-80% confluent were harvested. Proteins were then subjected to western blotting with the antibodies against S6K1, cyclin D1, cyclin E1, CDK2, CDK4, CDK6, p-Rb, and c-Myc proteins. GAPDH/β-Actin was used as the loading control. **H** Dual luciferase reporter assay showing the transcriptional activity of *CCNE1* promoter in S6K1-depleted MCF-7 cells. *P* value was calculated by Student’s *t*-test. **, *P* < 0.01. **I** c-Myc was re-expressed into S6K-depleted MCF-7 cells and the levels of cyclin E1 and p-Rb were measured by western blotting

Next, we investigated the mechanism by which *S6K1* amplification promotes resistance to CDK4/6 inhibitors. Given that cell proliferation may attribute to cell cycle progression, we examined the cell cycle distribution using flow cytometry. As shown in Fig. 5A, S6K1 knockdown increased the percentage of cells in the G1 phase, while exogenous expression of S6K1 decreased the percentage of cells in the G1 phase. To further unveil the key pathways affected by S6K1, we employed genome-wide RNA-sequencing (RNA-seq) to detect the S6K1 depletion-induced RNA expression alterations. In total, 826 significantly differentially expressed genes (DEGs) were identified, with 486 genes up regulated and 340 genes down regulated in S6K1-depleted MCF-7 cells (*P* < 0.05, log2Fold Change > 1 or < − 1, Supplementary Table S7). Kyoto Encyclopedia of Genes and Genomes (KEGG) analysis and Gene Ontology (GO) analysis were then conducted on these down regulated genes, and showed that cell proliferation-related pathways and cell cycle-related pathways were enriched (Fig. 5B and C). Meanwhile, protein chip detection also showed similar result (Supplementary Fig. 3F and G), further supporting the fact that knockdown of S6K1 in MCF-7 cells inhibited cell proliferation.

GSEA was further conducted by using the RNA-seq data and revealed that cell cycle phase and the E2F targets sets were significantly enriched (Fig. 5D; Supplementary Fig. S3H). Notably, the c-Myc targets also showed significant enrichment (Fig. 5D), indicating that the activation of c-Myc pathway may contribute to the cell cycle promotion caused by S6K1. Several cell cycle-related proteins were then examined in S6K1-depleted and overexpressed cells. As expected, the expression levels of CDK4, CDK6, cyclin D1, and cyclin E1, as well as the phosphorylation levels of Rb, were downregulated in S6K1 knockdown cells (Fig. 5E) but upregulated in the cells with exogenous expression of S6K1 (Fig. 5F), when compared to those in the control group.

Considering the results of GSEA and the literature reporting the translational activation of c-Myc by S6K1 [28–30], we detected the protein level of c-Myc and found that it was decreased in the S6K1-depleted cells but increased in the exogenous S6K1-expressed cells compared with the control group (Fig. 5G). Meanwhile, the mRNA level of cyclin E1 was significantly decreased, whereas c-Myc mRNA was not affected upon S6K1 depletion (Supplementary Fig. S3I and J).

Given that cyclin E1 was previously reported to be transcriptionally regulated by c-Myc [31], we confirmed the binding of c-Myc to *cyclin E1* promoter region in MCF-7 cells by applying the ChIP assay (Supplementary Fig. S3K). To detect whether cyclin E1 was transcriptionaly regulated in S6K1-depleted cells, luciferase reporter assay containing the *cyclin E1* promoter region was constructed. Analysis of the dual luciferase assay showed that depletion of S6K1 in MCF-7 cells significantly decreased the luciferase activity (Fig. 5H). Rescue experiment was performed to validate the c-Myc-mediated decrease of cyclin E1 in S6K1-depleted cells. After re-expression of c-Myc in S6K1-depleted cells, the levels of cyclin E1 and p-Rb were increased accordingly (Fig. 5I).

Collectively, these results indicated that S6K1 promoted cell proliferation by accelerating G1/S transition. Mechanically, S6K1 elevated the expression of cell cycle-related proteins such as cyclin E1, mainly through stimulating the c-Myc signaling pathway.

### S6K1 blockade increases palbociclib sensitivity in vitro and in vivo

**Fig. 6:**
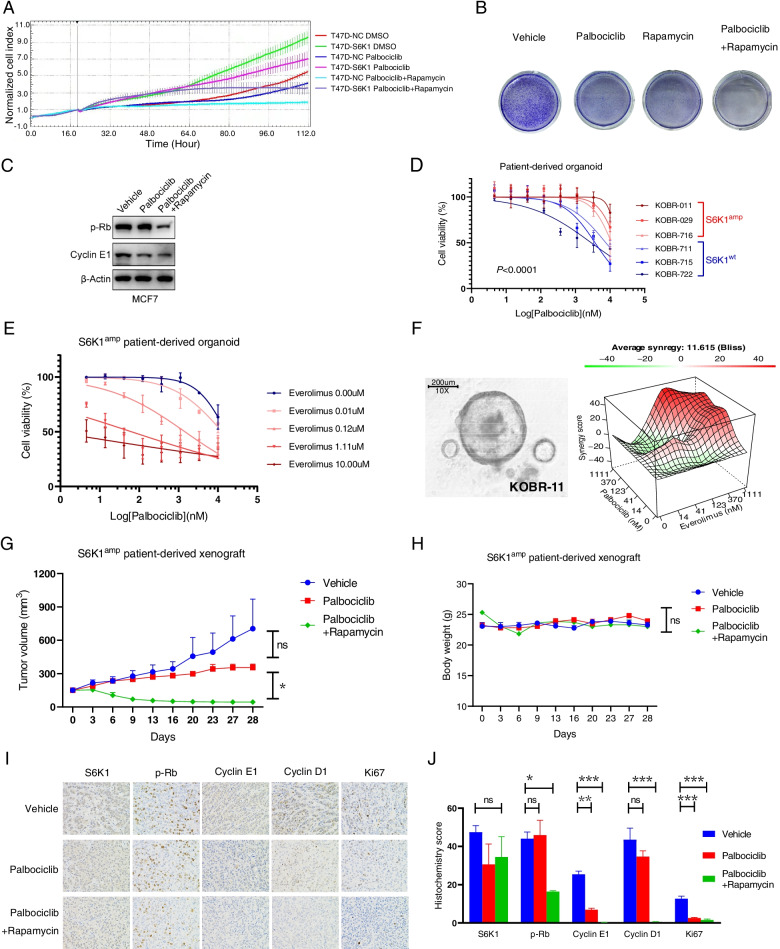
mTOR inhibitors can reverse resistance to palbociclib in vitro and in vivo. **A** xCELLigence system analysis of the proliferation of exogenous S6K1-expressed T47D cells or control cells treated with either vehicle, palbociclib (100 nM) or palbociclib (100 nM) plus mTOR inhibitor rapamycin (500 nM). **B** Clonogenic assay of MCF-7 cells treated with either vehicle, palbociclib (5 μM), rapamycin (1 μM) or combination. **C** The expression of cyclin E1 and p-Rb in MCF-7 cells treated as described in **B** was detected by western blotting. β-Actin was used as the loading control. **D** Three breast cancer patient-derived organoids (PDOs) with S6K1 amplification (KOBR-011, − 029, − 716) were identified and confirmed to be resistant to palbociclib. KOBR-711, − 715, and − 722 with wild type S6K1 were used as negative controls. **E** Addition of mTOR inhibitor everolimus to palbociclib showed greater inhibition of cell viability in the S6K1-amplificated PDO. **F** The landscape of the combination responses for palbociclib plus everolimus in the S6K1-amplificated PDO (KOBR-011) via the Bliss model. **G** In vivo tumour-growth measurement of S6K1-amplificated patient derived xenografts treated with either vehicle, palbociclib or palbociclib plus rapamycin for 28 days. Data are presented as the mean ± SEM; *P* value was calculated by Mixed-effects model. ns, not significant. *, *P* < 0.05. **H** Dynamic changes in body weight of mice as described in **G**. Data are presented as the mean ± SEM; *P* value was calculated by Mixed-effects model. ns, not significant. **I**, **J** Immunohistochemistry analysis of mouse tumour slices with the indicated antibodies. **I** Representative images. **J** Quantitative analysis. Data are presented as the mean ± SEM. *P* value was calculated by one-way ANOVA. ns, not significant. *, *P* < 0.05, **, *P* < 0.01, ***, *P* < 0.001

Given that S6K1 is directly activated by mTORC1 [32], we applied clinically available inhibitor of mTORC1 to assess whether palbociclib resistance could be reversed. Exogenous S6K1-expressed T47D cells were relatively resistant to palbociclib and this effect was completely reversed upon the addition of rapamycin (mTOR inhibitor, Fig. 6A). Similar results were obtained in MCF-7 cells (Fig. 6B, Supplementary Fig. S4A). Moreover, the combination of palbociclib with rapamycin significantly reduced the levels of p-Rb and cyclin E1 compared to palbociclib alone in MCF-7 cells (Fig. 6C).

To further investigate the efficacy of the combination therapy, we identified 3 organoids with amplified *S6K1* gene, KOBR-011, KOBR-029, and KOBR-716, from 32 organoids derived from patients with breast cancer (3/32, 9.4%, Fig. 1C). Compared with S6K1^wt^ PDOs (KOBR-711, KOBR-715, KOBR-722), organoids with *S6K1* gene amplification (S6K1^amp^) were significantly more resistant to palbociclib (*P* < 0.0001, Fig. 6D). The IC_50_ values of palbociclib were 15 μM, 15 μM and 11 μM in S6K1^amp^ PDOs, respectively, and 7 μM, 4 μM and 3 μM in S6K1^wt^ PDOs, respectively. S6K1^amp^ PDOs were resistant to both palbociclib and everolimus (mTOR inhibitor) as monotherapy, with IC_50_ values of 23 μM and 13 μM respectively (Supplementary Fig. S4B). However, in the presence of everolimus, a significant left shift of the dose–efficacy curve and a decrease in IC_50_ were observed in S6K1^amp^ PDOs (Fig. 6E). Upon the addition of 0.01 μM, 0.12 μM, 1.11 μM, and 10.00 μM everolimus, the IC_50_ of palbociclib decreased from 16 μM to 13 μM (*P* = 0.436), 1 μM (*P* = 0.046), 0.06 μM (*P* = 0.046), and 0.6 nM (*P* = 0.024), respectively. The synergistic effect of the combined regimen of palbociclib plus everolimus was then quantified in a S6K1^amp^ PDO KOBR-011 using the Bliss method. Synergy heatmaps showed that palbociclib combined with everolimus had a synergistic effect (red areas in the model graph) on inhibition of cell proliferation at a wide range of drug combination ratios (Supplementary Fig. S4C). The combination of palbociclib with everolimus showed an average synergy score of 11.6 (Fig. 6F). These data suggest that palbociclib in combination with everolimus exerts a synergistic effect in *S6K1*-amplified breast cancer PDO models.

To confirm the in vivo translatability of our findings, we next examined the effect of the dual combination in the PDX model with *S6K1* amplification. *S6K1*- amplificated tumours treated with palbociclib did not regress (*P* = 0.721, Fig. 6G), thus confirming the ability of *S6K1* amplification to confer resistance to palbociclib in vivo. Consistent with our findings in cell experiments and PDO studies, the combination of mTOR inhibitor with palbociclib significantly reverted this drug resistance (*P* = 0.021, Fig. 6G). On day 28, tumour growth inhibition rate was 63% in the palbociclib monotherapy group, and 119% in the palbociclib plus everolimus group (*P* = 0.024, Supplementary Fig. S4D). Importantly, the drug combinations didn’t cause significant weight loss (*P* = 0.695, Fig. 6H), indicating a lack of generalized toxicity. We next assessed the expression of S6K1 and cell cycle genes in tumour sections by IHC (Fig. 6I and J). All of these tumours with S6K1 gene amplification had high expression of S6K1. In these S6K1-overexpressed tumours, palbociclib monotherapy could not successfully downregulate the levels of cell cycle-related genes, such as p-Rb (*P* = 0.962) and cyclin D1 (*P* = 0.320). The addition of mTOR inhibitor to palbociclib markedly reduced p-Rb (*P* = 0.017) and cyclin D1 (*P* < 0.001) levels in these S6K1-overexpressed tumours. The level of cyclin E1 could be reduced by palbociclib monotherapy (*P* = 0.002), and furtherly decreased after adding the mTOR inhibitor (*P* < 0.001).

Together, these results suggest that S6K1plays a critical role in the resistance of CDK4/6 inhibitors, and that mTOR inhibitor could be considered to convert the resistance of CDK4/6 inhibitor caused by *S6K1* amplification.

## Discussion

To our knowledge, this study is the first to propose ctDNA-based *S6K1* amplification to determine innate resistance to CDK4/6 inhibitors in patients with ER^+^HER2^−^ MBC. The findings of in vitro and in vivo studies furtherly suggest that *S6K1* amplification-induced resistance to CDK4/6 inhibitors may mainly mediate by c-Myc signalling cascades that induce hyperactivation of cyclins/CDKs (Supplementary Fig. S5). Thus, patients with *S6K1* amplification may not be suitable for combined endocrine treatment with CDK4/6 inhibitors. For these patients, S6K1 blockade using upstream mTOR inhibitor could increase the sensitivity of breast cancer to CDK4/6 inhibitor.

Genotyping ctDNA is useful for detecting the panorama of genomic alterations, especially in clonal evolution for acquired drug resistance [12, 33]. Previous studies have also used ctDNA to search for genes related to innate resistance to CDK4/6 inhibitors, with much smaller panel size. In the PALOMA-3 cohort, by analysing the alteration of ctDNA in 17-mutated genes and 14-copy number altered genes, *P53* mutation and *FGFR1* amplification were identified to be associated with innate resistance to palbociclib [11]. The MONALEESA-7 study found a correlation between *CCND1* abnormalities and ribociclib resistance using a ctDNA panel of fewer than 600 genes [34]. To our knowledge, this study employed the largest ctDNA assay panel to date to search for biomarkers associated with innate resistance to CDK4/6 inhibitor. We analysed ctDNA data using a panel of 1021 genes, and successfully found that *S6K1* was a candidate for predicting innate resistance to CDK4/6 inhibitors in two cohorts of patients with MBC. *S6K1* amplification occurs in a relatively large fraction of CDK4/6 inhibitor- candidate patients (14% of patients with MBC, substantially in the ER^+^ subtype). Thus, we believe that patients with ER^+^ MBC would benefit from ctDNA analysis including *S6K1* amplification before initiating CDK4/6 inhibitor treatment. ctDNA testing can detect *S6K1* amplification as well as other mutations, thereby avoiding patients with drug resistance from receiving CDK4/6 inhibitor treatment.

Our study revealed that *S6K1* amplification was detected exclusively at the baseline before use of CDK4/6 inhibitors. *S6K1* amplification did not occur after the treatment of palbociclib, thereby indicating that *S6K1* amplification is not induced by palbociclib. Thus, *S6K1* amplification is related to primary drug resistance to palbociclib (innate resistance), instead of acquired resistance. A series of large-scale phase III clinical trials have conducted ctDNA analysis for CDK4/6 inhibition in MBC, but have failed to find a specific target that is clearly related to the resistance of CDK4/6 inhibitors [34, 35]. All these studies enrolled treatment-naïve patients or those with previously ≤1 line of endocrine therapy for MBC. However, in our study, we included heavily pre-treated patients with a median of 3 lines of endocrine treatment. The efficacy of palbociclib-based therapy in this population is relatively low, but it may provide more opportunities to identify biomarkers of innate resistance to CDK4/6 inhibition.

The *PI3K/AKT/mTOR* signalling pathway has been previously implicated in mediating early adaptation or acquired resistance to CDK4/6 inhibitors in combination with endocrine therapy [36–38]. Inhibition of CDK4/6 promotes the activation of AKT signalling in breast cancer cells, which results in cyclin D1 accumulation [37]. Thus, the gene abnormality of *PI3K/AKT/mTOR* related to CDK4/6 inhibition usually occurs after the initiation of CDK4/6 inhibitor (as acquired resistance) [36, 39]. Notably, our ctDNA analysis revealed that *S6K1* gene amplification, a downstream genetic abnormality that might be mutually exclusive to *PI3K* mutations, existed before the inhibition of CDK4/6, and is thus associated with innate drug resistance. Therefore, the use of ctDNA to detect *S6K1* gene amplification has clinical value in predicting palbociclib efficacy before treatment initiation, which is not possible for *PI3K* mutations associated with acquired resistance.

S6K1 activation regulates the translation of a subset of mRNA by phosphorylating its downstream ribosomal protein S6 and eIF4B [40–42]. A previous study in melanoma found that S6K1 inhibition can revert PIK3CA mutation-induced resistance to CDK4/6 inhibitor, while the specific molecular mechanism remained unknown [43]. In this study of breast cancer, our cell experiments revealed that overexpression of S6K1 increased the protein levels of c-Myc, thus activated the transcription of cyclin E1, which is consistent with findings of previous studies [28–31]. High cyclin E1 levels may contribute to resistance to CDK4/6 inhibitors by associating with CDK2 and subsequently promoting progression of the cell cycle from G1 to S phase [10, 37, 44]. Consistently, we observed that the inhibition of S6K1 activity by mTOR inhibitor was sufficient to increase the inhibitory effect of palbociclib on cell proliferation. PDX studies further confirmed that mTOR inhibitor could revert resistance to CDK4/6 inhibitor and downregulated the expression of cyclin E1. Thus, *S6K1* gene amplification plays an important role in cell cycle progression and resistance to CDK4/6 inhibitors.

Organoids formed from patient-derived cancer cells can recapitulate patient responses in the clinic and are now used for evaluating drug sensitivity in various types of cancer, including breast cancer [23, 45]. Thus, we confirmed the relationship between *S6K1* amplification and palbociclib resistance in a PDO model system. Similar to the breast cancer cell lines, we observed palbociclib resistance in PDOs with *S6K1* amplification. Furthermore, we demonstrated that the combination of palbociclib plus mTOR inhibitor everolimus showed higher sensitivity than monotherapy in PDOs with *S6K1* amplification. This effect appeared to be due to the synergistic effect between these two drugs, demonstrated via the Bliss method. Everolimus in combination with several endocrine therapies has shown significant efficacy benefits with a tolerable safety profile in clinical trials and clinical routine [46, 47]. Clinical trials evaluating the safety and tolerability of the addition of everolimus to the combination of CDK4/6 inhibitor plus exemestane in patients with ER^+^ HER2^−^ MBC have been launched (NCT02732119, NCT01857193).

## Conclusions

In summary, *S6K1* amplification occurred frequently in patients with ER^+^ MBC, could be detected by ctDNA analysis, and was closely related to the innate resistance of CDK4/6 inhibitors. *S6K1* gene amplification resulted in increased expression of c-Myc and subsequently elevated expression of cyclin E1, thus played an important role in regulating cell cycle progression. Based on these results, we propose mTOR inhibitors in combination with CDK4/6 inhibitors as a potential therapeutic strategy in ER^+^HER2^−^ breast cancers with *S6K1* amplification.

## Supplementary Information

**Additional file 1: Supplementary Table S1.** List of target region genes in the 1021-gene panel.

**Additional file 2: Supplementary Table S2.** The copy number variation of *S6K1* gene (S6K1:CEP17 via ddPCR) in patients with low S6K1 expression.

**Additional file 3: Supplementary Table S3.** Baseline clinical and pathological characteristics of patients in the discovery cohort.

**Additional file 4: Supplementary Table S4.** Detected molecular alterations and their mutant allele frequencies at baseline in the discovery cohort.

**Additional file 5: Supplementary Table S5.** The correlations between the mRNA levels of S6K1 and other PI3K/AKT/mTOR pathway genes in the breast cancer cohort of the TCGA PanCancer Atlas, using data from the cBioPortal.

**Additional file 6: Supplementary Table S6.** Clinical characteristics of patients received palbociclib treatment in the validation cohort.

**Additional file 7: Supplementary Table S7.** The list of significantly differentially expressed genes in S6K1-depleted MCF-7 cells revealed by RNA-seq.

**Additional file 8: Supplementary Fig. S1.** Analysis of genetic abnormalities in plasma samples from patients receiving palbociclib treatment in the discovery cohort (related to Fig. 1). (A) The mutated locus of *PIK3CA*, *TP53*, and *ESR1* genes in patients with or without innate resistance to palbociclib. (B) Diverse PI3K-pathway activating events observed in 19 patients. (C) The detected molecular alterations in functional genes of patients with or without innate resistance to palbociclib. (D) The detected molecular alterations at the baseline and after disease progression. IR: innate resistant; CB: clinical benefit. (E) The analysis of interactions between high-frequency altered genes in patients with or without innate resistance to palbociclib. S6K1 appears to be mutually exclusive with PIK3CA gene in patients with innate resistance to palbociclib.

**Additional file 9: Supplementary Fig. S2. ***S6K1* gene amplification is correlated with high expression of S6K1 and cell cycle-related genes in the TCGA cohort (related to Fig. 2). (A) Copy number variation of *S6K1 (RPS6KB1)* gene in patients with metastatic or primary breast cancer, based on cBioPortal. (B) Analysis of correlations between *S6K1 (RPS6KB1)* gene amplification and S6K1 mRNA expression (960 samples with data in both profiles) as well as S6K1 mRNA and protein expression (780 samples with data in both profiles) in breast cancer, based on cBioPortal. (C) The signalling pathways that are affected by high expression of S6K1 analysed by GSEA. (D) Analysis of correlations between S6K1 and cell cycle-related gene expression in BRCA cohort, based on GEPIA. *P* value was calculated by Spearman’s correlation.

**Additional file 10: Supplementary Fig. S3.** Related to Figs. 4 and 5. (A) The copy number and mRNA levels of S6K1 in breast cancer cell lines were obtained from Cancer Cell Line Encyclopedia (CCLE) (https://portals.broadinstitute.org/ccle). Y-axis represents mRNA expression and X-axis represents copy number. (B, C) MCF-7 cells were transfected with S6K1 siRNAs (siS6K1-1/− 2/− 3) or non-sense control siRNA (siNC). The RNAi efficiency was validated using RT-qPCR (B) and western blotting (C). (D, E) Stable exogenous S6K1-expressed T47D cells and control cells were validated using RT-qPCR (D) and western blotting (E). (F, G) KEGG enrichment of signalling pathways of phosphorylated (F) or non-phosphorylated (G) proteins detected by protein chip in S6K1-depleted MCF-7 cells. (H) GSEA plot of RNA-seq data from S6K1-depleted MCF-7 cells for cell cycle phase. (I, J) MCF-7 cells were transfected with S6K1 siRNA pool or non-sense control siRNA. The mRNA levels of c-Myc and cyclin E1 were measured using RT-qPCR. (K) ChIP assay for the binding ability of c-Myc to the *cyclin E1* promoter element in MCF-7 cells. *P* value was calculated by Student’s *t*-test. *, *P* < 0.05, **, *P* < 0.01, ***, *P* < 0.001.

**Additional file 11: Supplementary Fig. S4.** Related to Fig. 6. (A) xCELLigence system analysis of the proliferation of MCF-7 cells treated with either vehicle, palbociclib (5 μM), rapamycin (1 μM) or combination. (B) S6K1-amplified PDOs from breast cancers showed limited response to everolimus or palbociclib as monotherapy. Data are presented as the mean ± SEM; *P* value was calculated by 2-way ANOVA. (C) Dose–response matrix for the effect of palbociclib plus mTOR inhibitor (everolimus) in a S6K1-amplificated organoid derived from breast cancer patient. (D) Tumour growth inhibition (TGI) curve in S6K1-amplificated xenograft derived from breast cancer patient. Data are presented as the mean ± SEM; *P* value was calculated by Mixed-effects model. *, *P* < 0.05. TGI was calculated using the following formula: TGI = 1 − (tumour volume change of the treated group relative to day 0)/(tumour volume change of the control group relative to day 0).

**Additional file 12: Supplementary Fig. S5.** Schematic representation of the proposed signal transduction pathways in S6K1-mediated cell proliferation and resistance to CDK4/6 inhibitors. Figures made in ©BioRender-biorender.com.

## Data Availability

All data generated or analysed during this study are included in this published article.

## References

[1] Chen W, Zheng R, Baade PD, Zhang S, Zeng H, Bray F, Jemal A, Yu XQ, He J (2016). Cancer statistics in China, 2015. CA Cancer J Clin.

[2] Sung H, Ferlay J, Siegel RL, Laversanne M, Soerjomataram I, Jemal A, Bray F (2021). Global cancer statistics 2020: GLOBOCAN estimates of incidence and mortality worldwide for 36 cancers in 185 countries. CA Cancer J Clin.

[3] Ma F, Wu J, Fu L, Li A, Lan B, Chen K, Di J, Jiang Y, Li J, Li N (2021). Interpretation of specification for breast cancer screening, early diagnosis, and treatment management in Chinese women. J Natl Cancer Center.

[4] Gong Y, Liu YR, Ji P, Hu X, Shao ZM (2017). Impact of molecular subtypes on metastatic breast cancer patients: a SEER population-based study. Sci Rep.

[5] Finn RS, Martin M, Rugo HS, Jones S, Im SA, Gelmon K, Harbeck N, Lipatov ON, Walshe JM, Moulder S (2016). Palbociclib and letrozole in advanced breast cancer. N Engl J Med.

[6] Turner NC, Slamon DJ, Ro J, Bondarenko I, Im SA, Masuda N, Colleoni M, DeMichele A, Loi S, Verma S (2018). Overall survival with palbociclib and fulvestrant in advanced breast cancer. N Engl J Med.

[7] Im SA, Lu YS, Bardia A, Harbeck N, Colleoni M, Franke F, Chow L, Sohn J, Lee KS, Campos-Gomez S (2019). Overall survival with ribociclib plus endocrine therapy in breast cancer. N Engl J Med.

[8] Fassl A, Geng Y, Sicinski P (2022). CDK4 and CDK6 kinases: from basic science to cancer therapy. Science.

[9] Finn RS, Liu Y, Zhu Z, Martin M, Rugo HS, Diéras V, Im SA, Gelmon KA, Harbeck N, Lu DR (2020). Biomarker analyses of response to cyclin-dependent kinase 4/6 inhibition and endocrine therapy in women with treatment-naïve metastatic breast cancer. Clin Cancer Res.

[10] Turner NC, Liu Y, Zhu Z, Loi S, Colleoni M, Loibl S, et al. Cyclin E1 expression and palbociclib efficacy in previously treated hormone receptor-positive metastatic breast cancer. J Clin Oncol. 2019. 10.1200/JCO.18.00925. 10.1200/JCO.18.00925 PMC6506420 30807234

[11] O'Leary B, Cutts RJ, Huang X, Hrebien S, Liu Y, André F, Loibl S, Loi S, Garcia-Murillas I, Cristofanilli M (2021). Circulating tumor DNA markers for early progression on fulvestrant with or without palbociclib in ER+ advanced breast cancer. J Natl Cancer Inst.

[12] O'Leary B, Cutts RJ, Liu Y, Hrebien S, Huang X, Fenwick K, André F, Loibl S, Loi S, Garcia-Murillas I (2018). The genetic landscape and clonal evolution of breast cancer resistance to palbociclib plus fulvestrant in the PALOMA-3 trial. Cancer Discov.

[13] Andre F, Su F, Solovieff N, Arteaga CL, Hortobagyi GN, Chia SKL, Neven P, Bardia A, Tripathy D, Lu Y-S (2020). Pooled ctDNA analysis of the MONALEESA (ML) phase III advanced breast cancer (ABC) trials. J Clin Oncol.

[14] Sanz-Garcia E, Zhao E, Bratman SV, Siu LL (2022). Monitoring and adapting cancer treatment using circulating tumor DNA kinetics: current research, opportunities, and challenges. Sci Adv.

[15] Wang DS, Liu ZX, Lu YX, Bao H, Wu X, Zeng ZL, Liu Z, Zhao Q, He CY, Lu JH (2019). Liquid biopsies to track trastuzumab resistance in metastatic HER2-positive gastric cancer. Gut.

[16] Yi Z, Ma F, Rong G, Liu B, Guan Y, Li J, Sun X, Wang W, Guan X, Mo H (2021). The molecular tumor burden index as a response evaluation criterion in breast cancer. Signal Transduct Target Ther.

[17] Yi Z, Rong G, Guan Y, Li J, Chang L, Li H, Liu B, Wang W, Guan X, Ouyang Q (2020). Molecular landscape and efficacy of HER2-targeted therapy in patients with HER2-mutated metastatic breast cancer. NPJ Breast Cancer.

[18] Lefebvre C, Bachelot T, Filleron T, Pedrero M, Campone M, Soria JC, Massard C, Lévy C, Arnedos M, Lacroix-Triki M (2016). Mutational profile of metastatic breast cancers: a retrospective analysis. PLoS Med.

[19] Wagle N, Painter C, Krevalin M, Oh C, Anderka K, Larkin K, Lennon N, Dillon D, Frank E, Winer EP (2016). The metastatic breast cancer project: a national direct-to-patient initiative to accelerate genomics research. J Clin Oncol.

[20] McCarthy DJ, Chen Y, Smyth GK (2012). Differential expression analysis of multifactor RNA-Seq experiments with respect to biological variation. Nucleic Acids Res.

[21] Yu G, Wang LG, Han Y, He QY (2012). clusterProfiler: an R package for comparing biological themes among gene clusters. Omics.

[22] Ye S, Li C, Zheng X, Huang W, Tao Y, Yu Y, Yang L, Lan Y, Ma L, Bian S, Du W (2022). OsciDrop: a versatile deterministic droplet generator. Anal Chem.

[23] Sachs N, de Ligt J, Kopper O, Gogola E, Bounova G, Weeber F, Balgobind AV, Wind K, Gracanin A, Begthel H (2018). A living biobank of breast cancer organoids captures disease heterogeneity. Cell.

[24] He L, Kulesskiy E, Saarela J, Turunen L, Wennerberg K, Aittokallio T, Tang J (2018). Methods for high-throughput drug combination screening and synergy scoring. Methods Mol Biol.

[25] Zhao W, Sachsenmeier K, Zhang L, Sult E, Hollingsworth RE, Yang H (2014). A new bliss Independence model to analyze drug combination data. J Biomol Screen.

[26] Győrffy B (2021). Survival analysis across the entire transcriptome identifies biomarkers with the highest prognostic power in breast cancer. Comput Struct Biotechnol J.

[27] Barretina J, Caponigro G, Stransky N, Venkatesan K, Margolin AA, Kim S, Wilson CJ, Lehár J, Kryukov GV, Sonkin D (2012). The cancer cell line encyclopedia enables predictive modelling of anticancer drug sensitivity. Nature.

[28] Csibi A, Lee G, Yoon SO, Tong H, Ilter D, Elia I, Fendt SM, Roberts TM, Blenis J (2014). The mTORC1/S6K1 pathway regulates glutamine metabolism through the eIF4B-dependent control of c-Myc translation. Curr Biol.

[29] Foster DA, Yellen P, Xu L, Saqcena M (2010). Regulation of G1 cell cycle progression: distinguishing the restriction point from a nutrient-sensing cell growth checkpoint(s). Genes Cancer.

[30] Ma XM, Blenis J (2009). Molecular mechanisms of mTOR-mediated translational control. Nat Rev Mol Cell Biol.

[31] Obaya AJ, Mateyak MK, Sedivy JM (1999). Mysterious liaisons: the relationship between c-Myc and the cell cycle. Oncogene.

[32] Hay N, Sonenberg N (2004). Upstream and downstream of mTOR. Genes Dev.

[33] Condorelli R, Spring L, O'Shaughnessy J, Lacroix L, Bailleux C, Scott V, Dubois J, Nagy RJ, Lanman RB, Iafrate AJ (2018). Polyclonal RB1 mutations and acquired resistance to CDK 4/6 inhibitors in patients with metastatic breast cancer. Ann Oncol.

[34] Bardia A, Su F, Solovieff N, Im SA, Sohn J, Lee KS, Campos-Gomez S, Jung KH, Colleoni M, Vázquez RV (2021). Genomic profiling of premenopausal HR+ and HER2- metastatic breast cancer by circulating tumor DNA and association of genetic alterations with therapeutic response to endocrine therapy and ribociclib. JCO Precis Oncol.

[35] Cristofanilli M, Turner NC, Bondarenko I, Ro J, Im SA, Masuda N, Colleoni M, DeMichele A, Loi S, Verma S (2016). Fulvestrant plus palbociclib versus fulvestrant plus placebo for treatment of hormone-receptor-positive, HER2-negative metastatic breast cancer that progressed on previous endocrine therapy (PALOMA-3): final analysis of the multicentre, double-blind, phase 3 randomised controlled trial. Lancet Oncol.

[36] Wander SA, Cohen O, Gong X, Johnson GN, Buendia-Buendia J, Lloyd MR, Kim D, Luo F, Mao P, Helvie K (2020). The genomic landscape of intrinsic and acquired resistance to cyclin-dependent kinase 4/6 inhibitors in patients with hormone receptor positive metastatic breast cancer. Cancer Discov.

[37] Herrera-Abreu MT, Palafox M, Asghar U, Rivas MA, Cutts RJ, Garcia-Murillas I, Pearson A, Guzman M, Rodriguez O, Grueso J (2016). Early adaptation and acquired resistance to CDK4/6 inhibition in estrogen receptor-positive breast cancer. Cancer Res.

[38] Michaloglou C, Crafter C, Siersbaek R, Delpuech O, Curwen JO, Carnevalli LS, Staniszewska AD, Polanska UM, Cheraghchi-Bashi A, Lawson M (2018). Combined inhibition of mTOR and CDK4/6 is required for optimal blockade of E2F function and long-term growth inhibition in estrogen receptor-positive breast cancer. Mol Cancer Ther.

[39] O'Leary B, Hrebien S, Morden JP, Beaney M, Fribbens C, Huang X, Liu Y, Bartlett CH, Koehler M, Cristofanilli M (2018). Early circulating tumor DNA dynamics and clonal selection with palbociclib and fulvestrant for breast cancer. Nat Commun.

[40] Saxton RA, Sabatini DM (2017). mTOR signaling in growth, metabolism, and disease. Cell.

[41] Bahrami BF, Ataie-Kachoie P, Pourgholami MH, Morris DL (2014). p70 ribosomal protein S6 kinase (Rps6kb1): an update. J Clin Pathol.

[42] Holz MK, Ballif BA, Gygi SP, Blenis J (2005). mTOR and S6K1 mediate assembly of the translation preinitiation complex through dynamic protein interchange and ordered phosphorylation events. Cell.

[43] Romano G, Chen PL, Song P, McQuade JL, Liang RJ, Liu M, Roh W, Duose DY, Carapeto FCL, Li J (2018). A preexisting rare PIK3CA(E545K) subpopulation confers clinical resistance to MEK plus CDK4/6 inhibition in NRAS melanoma and is dependent on S6K1 signaling. Cancer Discov.

[44] Chu C, Geng Y, Zhou Y, Sicinski P (2021). Cyclin E in normal physiology and disease states. Trends Cell Biol.

[45] Vlachogiannis G, Hedayat S, Vatsiou A, Jamin Y, Fernández-Mateos J, Khan K, Lampis A, Eason K, Huntingford I, Burke R (2018). Patient-derived organoids model treatment response of metastatic gastrointestinal cancers. Science.

[46] Bachelot T, Bourgier C, Cropet C, Ray-Coquard I, Ferrero JM, Freyer G, Abadie-Lacourtoisie S, Eymard JC, Debled M, Spaëth D (2012). Randomized phase II trial of everolimus in combination with tamoxifen in patients with hormone receptor-positive, human epidermal growth factor receptor 2-negative metastatic breast cancer with prior exposure to aromatase inhibitors: a GINECO study. J Clin Oncol.

[47] Jerusalem G, de Boer RH, Hurvitz S, Yardley DA, Kovalenko E, Ejlertsen B, Blau S, Özgüroglu M, Landherr L, Ewertz M (2018). Everolimus plus exemestane vs everolimus or capecitabine monotherapy for estrogen receptor-positive, HER2-negative advanced breast cancer: the BOLERO-6 randomized clinical trial. JAMA Oncol.

