# Supplementary material for: S6K1 amplification confers innate resistance to CDK4/6 inhibitors through activating c-Myc pathway in patients with estrogen receptor-positive breast cancer
Source: Mol Cancer. 2022 Aug 30;21:171. doi: 10.1186/s12943-022-01642-5 (PMC9426012; doi:10.1186/s12943-022-01642-5)
Supplement: Supplementary file 1 — Additional file 1: Supplementary Table S1. List of target region genes in the 1021-gene panel. [file 12943_2022_1642_MOESM1_ESM.docx]

| **Supplementary Table S1. List of target region genes in the 1021-gene panel.** | | | | |
| --- | --- | --- | --- | --- |
| ABCA10 | CSNK1E | HSPD1 | NUP210 | SLC2A2 |
| ABCA8 | CSPP1 | HYDIN | NUTM1 | SLC30A5 |
| ABCB7 | CTCF | IBSP | NWD1 | SLC35B2 |
| ABCC8 | CTIF | IDH1 | NXF1 | SLC35B4 |
| ABCF2 | CTNNA2 | IDH2 | NXF5 | SLC38A4 |
| ABL1 | CTNNB1 | IFT172 | OBP2A | SLC38A5 |
| ABL2 | CTSF | IGF1R | OBP2B | SLC43A1 |
| ACE | CYP2A13 | IGSF9 | OCA2 | SLC45A1 |
| ACER2 | CYP3A4 | IKBKAP | ODZ3 | SLC4A10 |
| ACOT11 | CYP4A11 | IKBKE | OR2T4 | SLC4A4 |
| ACPP | CYTH4 | IL11RA | OR4A15 | SLC5A1 |
| ACSL1 | DCLK2 | IL13RA2 | OR4C6 | SLC6A5 |
| ACSM5 | DCST1 | IL1RAPL1 | OR5L2 | SLC8A1 |
| ACSS3 | DDB1 | IL27RA | OR6F1 | SLCO1B7 |
| ACTL6B | DDR1 | IL7R | OSBPL10 | SLCO5A1 |
| ADAM23 | DDR2 | IMPG1 | OTOA | SMARCA4 |
| ADAM33 | DDX24 | INHBA | OTOGL | SMARCB1 |
| ADAMTS12 | DDX3X | INPP4B | OVCH1 | SMO |
| ADAMTS16 | DEPDC4 | INPP5J | P4HB | SMTN |
| ADAMTS19 | DGKK | IQCA1 | PABPC4 | SNTG1 |
| ADAMTS20 | DHCR24 | IRS2 | PACS2 | SORCS3 |
| ADAMTS5 | DHDDS | ITFG2 | PAEP | SPAG16 |
| ADAMTSL1 | DHX9 | ITGA8 | PAGE1 | SPATA13 |
| ADD2 | DIAPH1 | ITGA9 | PALB2 | SPG20 |
| AGMAT | DKC1 | ITIH1 | PARK2 | SPINT1 |
| AGTPBP1 | DLST | ITLN2 | PARP4 | SPPL2A |
| AHCTF1 | DMD | ITM2A | PCK2 | SPPL3 |
| AK5 | DMXL1 | ITPKB | PCLO | SPRED1 |
| AKR1B10 | DMXL2 | ITPR1 | PCNT | SPTA1 |
| AKR1C1 | DNAH10 | JAK1 | PCNXL2 | SRC |
| AKT1 | DNAH5 | JAK2 | PCSK5 | SRRT |
| AKT2 | DNAH9 | JAK3 | PCYT1A | SSBP3 |
| AKT3 | DNAJC11 | KCNAB2 | PDCD6 | SSH2 |
| ALDH1A3 | DNAJC9 | KCNH6 | PDE1C | SSPO |
| ALDH2 | DNMT3A | KCNQ2 | PDE2A | ST18 |
| ALG5 | DNTTIP1 | KDM4A | PDE4DIP | ST6GALNAC1 |
| ALK | DOCK11 | KDM6A | PDGFRA | STAG2 |
| ALX4 | DOCK3 | KDR | PDGFRB | STAT1 |
| AMOT | DOT1L | KEAP1 | PDIA5 | STAT3 |
| ANK2 | DPP10 | KIAA0195 | PDILT | STAT4 |
| ANKRD13D | DPP4 | KIAA0226 | PDK1 | STAT6 |
| ANKRD20A4 | DRGX | KIAA0319 | PDRG1 | STK11 |
| ANKRD27 | DUOX1 | KIAA0922 | PEX6 | STK11IP |
| ANKRD28 | DYSF | KIAA1191 | PGAP1 | STK31 |
| ANKRD30A | DZANK1 | KIAA1199 | PHACTR3 | STX3 |
| ANKRD30B | ECHDC1 | KIAA1211L | PHF20L1 | SULT1A4 |
| ANKRD36B | EDN1 | KIF13A | PHYH | SUPT5H |
| ANO2 | EEF1A1 | KIF1B | PI4KB | SUPT6H |
| AP1B1 | EFCAB5 | KIF26B | PIK3CA | SYCP2L |
| AP1G2 | EFCAB6 | KIF5B | PIK3CB | SYK |
| AP3B1 | EFCAB7 | KIFAP3 | PIK3R1 | SYNE1 |
| APAF1 | EFHA2 | KIFC1 | PIK3R2 | SYNE2 |
| APC | EFNA5 | KIR2DL3 | PIP4K2C | SYNJ2 |
| APLP2 | EGFR | KIR3DL3 | PIP5K1C | TAF1B |
| APMAP | EIF1AX | KIT | PIWIL1 | TAF6 |
| APPL2 | EIF2B5 | KLHL1 | PKD1L2 | TARBP1 |
| AQP12A | EIF2C2 | KLHL14 | PKHD1 | TBC1D1 |
| AR | EIF3E | KLK1 | PKLR | TBC1D21 |
| ARAF | EIF3I | KMT2B | PLAC8 | TBC1D3 |
| ARFGAP1 | EIF4ENIF1 | KMT2C | PLCB4 | TBC1D5 |
| ARFRP1 | EIF4H | KRAS | PLCZ1 | TBL1X |
| ARHGAP35 | ELAVL3 | KRT2 | PLEC | TBP |
| ARHGAP40 | ELL3 | KRT9 | PLK2 | TBX15 |
| ARHGEF1 | EMID2 | KRTAP5-5 | PLOD3 | TBX22 |
| ARHGEF7 | ENPP2 | KTN1 | PLXNA1 | TBX3 |
| ARNTL | ENTPD6 | L3MBTL1 | PMS1 | TCF20 |
| ARPC4-TTLL3 | EPB41L2 | LARP1 | PMS2 | TCF4 |
| ASH2L | EPB41L4B | LCN10 | POLDIP2 | TCP10 |
| ASTN1 | EPHA2 | LCT | POLE | TCP11 |
| ASXL2 | EPHA3 | LCTL | POLR2J | TEK |
| ATAD2B | EPHA5 | LETM1 | POLR3B | TERT |
| ATG9B | EPHB1 | LGALS13 | POLR3GL | TESC |
| ATM | EPS8L3 | LILRB3 | POLRMT | TEX35 |
| ATP10B | ERBB2 | LILRB4 | POM121L12 | TFDP1 |
| ATP10D | ERBB3 | LIPN | POTEG | TGDS |
| ATP12A | ERBB4 | LMAN1L | PPA1 | TGM2 |
| ATP2C1 | ERCC1 | LMBR1L | PPDPF | TGM5 |
| ATP6V0A2 | ERG | LPCAT4 | PPEF1 | THBS2 |
| ATP8B2 | ESD | LPHN3 | PPFIBP2 | THEM5 |
| ATR | ESR1 | LRBA | PPIL2 | THOC1 |
| ATXN2 | ETNK2 | LRP1B | PPP1R17 | THSD7A |
| ATXN7L2 | ETV6 | LRP2 | PPP4R4 | THSD7B |
| AURKA | EXOC4 | LRP4 | PQBP1 | TIMD4 |
| AURKB | EXOC5 | LRRC16B | PREB | TIMM44 |
| AXL | EXOC6 | LRRC2 | PREX2 | TIMP3 |
| BAP1 | EXOC7 | LRRC7 | PRKAA1 | TJP3 |
| BAX | EXTL3 | LRRC72 | PRKACA | TLE1 |
| BBS9 | EYA4 | LRRD1 | PRKAG3 | TLL1 |
| BCAS1 | EZH2 | LRRFIP2 | PRKCD | TMC2 |
| BCAS2 | F8 | LRSAM1 | PRKDC | TMED8 |
| BCL2 | F9 | LTBP1 | PRKX | TMEM104 |
| BCL2L11 | FAH | LUC7L2 | PRRX1 | TMEM120B |
| BCR | FAM114A2 | LUZP4 | PRSS1 | TMEM132D |
| BLOC1S1 | FAM131B | MAEL | PRUNE | TMEM145 |
| BMPR1B | FAM135B | MAGI1 | PSG2 | TMEM247 |
| BRAF | FAM13C | MAN2A1 | PSG5 | TMEM80 |
| BRCA1 | FAM157B | MAP2 | PSIP1 | TMEM87A |
| BRCA2 | FAM177B | MAP2K1 | PSMB1 | TMPRSS2 |
| BRD2 | FAM21A | MAP2K2 | PSMB5 | TMTC4 |
| BRD3 | FAM3A | MAP2K4 | PSMC4 | TMX3 |
| BRD4 | FAM49A | MAP3K1 | PSMC6 | TNFAIP6 |
| BRF1 | FAM49B | MAP4K1 | PSTPIP1 | TNFSF4 |
| BRSK2 | FAM5C | MAPK1 | PTBP3 | TNN |
| BRWD3 | FAM86B1 | MAPK3 | PTCD3 | TNNT1 |
| BSG | FAN1 | MAPKAPK3 | PTCH1 | TNR |
| BTK | FANCC | MAPRE3 | PTCH2 | TNS3 |
| BTNL3 | FASTK | MAST1 | PTEN | TOP1 |
| BTRC | FAT1 | MBIP | PTGES3L-AARSD1 | TP53 |
| C11orf30 | FATE1 | MBTPS2 | PTGS2 | TP53BP1 |
| C12orf5 | FBN2 | MCF2L2 | PTPLAD1 | TPCN1 |
| C19orf38 | FBXW7 | MCL1 | PTPN11 | TPH2 |
| C1orf112 | FCGR2A | MCOLN2 | PTPN13 | TPMT |
| C1orf35 | FCGR2B | MDGA2 | PTPRA | TPTE |
| C1QA | FCGR3A | MDM2 | PTPRD | TRIM33 |
| C1S | FDCSP | MDM4 | PTPRM | TRIM51 |
| C20orf112 | FGFR1 | MDN1 | PYHIN1 | TRIM58 |
| C2orf47 | FGFR2 | MED12 | QRICH2 | TRIML1 |
| C2orf62 | FGFR3 | MED23 | RAB1B | TRIO |
| C7orf53 | FGFR4 | MEFV | RAB3GAP2 | TRIP11 |
| C9orf114 | FLCN | MET | RAB6A | TRMT112 |
| C9orf43 | FLNC | METTL14 | RAC2 | TRPC5 |
| CACNA1A | FLOT2 | METTL5 | RAF1 | TRUB1 |
| CACNA1D | FLT1 | MGAM | RALBP1 | TSC1 |
| CACNA1E | FLT3 | MICALL1 | RAPGEF2 | TSC2 |
| CADM2 | FLT3LG | MID1 | RARA | TSGA10 |
| CAMKK1 | FLT4 | MIER2 | RARB | TSKS |
| CAPRIN1 | FMN2 | MITF | RASEF | TSPAN12 |
| CARS | FMNL3 | MLH1 | RB1 | TSR2 |
| CARS2 | FNDC4 | MLH3 | RBM6 | TTF2 |
| CASC4 | FNIP2 | MLL3 | RBMX | TTN |
| CASP8 | FOLH1 | MLPH | RCC1 | TUBA3C |
| CASP8AP2 | FOXA1 | MORC1 | REC8 | TUBGCP4 |
| CASQ2 | FOXJ2 | MORN1 | REG1B | TUBGCP5 |
| CATSPER2 | FOXL2 | MPL | RELN | TYK2 |
| CBFB | FRG1 | MRPL1 | RERE | TYRP1 |
| CBL | FRG2B | MRPL24 | RET | U2AF1 |
| CBX4 | FRMD4A | MRPS18B | RFWD2 | U2AF2 |
| CCDC155 | FRMPD2 | MS4A1 | RFX3 | UBASH3A |
| CCDC159 | FRMPD4 | MSH2 | RHEB | UBE2Q1 |
| CCDC17 | FSD2 | MSH3 | RHOA | UBE4B |
| CCND1 | FSHR | MSH6 | RICTOR | UCHL3 |
| CCND2 | FUBP1 | MSI1 | RNF215 | UCK2 |
| CCND3 | FUNDC1 | MTA2 | RNF219 | UGT8 |
| CCNE1 | GAB2 | MTM1 | RNF43 | ULK3 |
| CCT3 | GAB3 | MTOR | ROCK1 | UMOD |
| CCT6B | GABRD | MTR | ROS1 | UNC13A |
| CD1E | GAD2 | MTTP | RPL22 | UNC13D |
| CD274 | GALNT13 | MUC5B | RPL36A | UNC5D |
| CD300LF | GALNT14 | MUS81 | RPS5 | USP12 |
| CD5L | GATA3 | MYB | RPS6KA1 | USP34 |
| CD9 | GFRAL | MYBPC2 | RPS6KB1 | USP39 |
| CD97 | GIGYF1 | MYC | RPTOR | USP45 |
| CD99 | GINS4 | MYCBP2 | RPUSD4 | USP48 |
| CDH1 | GIPR | MYD88 | RREB1 | VAV1 |
| CDH18 | GKN2 | MYH15 | RRP7A | VEGFA |
| CDH24 | GLB1L3 | MYH2 | RUNDC3A | VEZF1 |
| CDH26 | GLYR1 | MYH4 | RUNX1 | VHL |
| CDK11A | GMDS | MYH8 | RYR2 | VILL |
| CDK12 | GNA11 | MYH9 | RYR3 | VIT |
| CDK13 | GNAQ | MYL5 | SAFB2 | VPS13A |
| CDK14 | GNAS | MYL6 | SAG | VPS33B |
| CDK18 | GNPTAB | MYLK2 | SAGE1 | VSIG4 |
| CDK19 | GOLGA4 | MYO3A | SAMD8 | WAS |
| CDK4 | GPAT2 | MYOM1 | SCN10A | WASL |
| CDK6 | GPATCH2 | NACAD | SCN3A | WDR44 |
| CDK8 | GPR114 | NARF | SCN7A | WDR52 |
| CDKN1A | GPR125 | NAT10 | SCN9A | WDR62 |
| CDKN1B | GPR133 | NAV3 | SDK2 | WDR66 |
| CDKN2A | GPR144 | NBPF1 | SEC14L4 | WDR72 |
| CDKN2B | GPS2 | NBPF10 | SEC24B | WDTC1 |
| CDS1 | GRIA3 | NCF2 | SEH1L | WLS |
| CEACAM20 | GRIK2 | NCKAP1 | SELP | WSCD2 |
| CECR2 | GUCY1A3 | NCOR1 | SEMA6A | WWP2 |
| CELA2B | GUCY2C | NCOR2 | SEPT12. | XBP1 |
| CGN | GYLTL1B | NEK5 | SERPINA7 | XPO1 |
| CHD3 | HAAO | NELL1 | SETD1B | XPO4 |
| CHD4 | HAP1 | NF1 | SETD2 | XPO5 |
| CHD6 | HAUS5 | NF2 | SF1 | XRCC1 |
| CHEK1 | HAUS6 | NFE2L2 | SF3B1 | ZAP70 |
| CHEK2 | HCN1 | NIPBL | SF3B14 | ZBTB8OS |
| CHI3L1 | HDAC1 | NLGN3 | SF3B3 | ZC3H13 |
| CISD3 | HDAC4 | NLRC3 | SGCZ | ZC3H7B |
| CLCN7 | HDAC6 | NLRP4 | SGIP1 | ZDHHC11 |
| CLEC16A | HEATR7B2 | NMI | SGK1 | ZFC3H1 |
| CLINT1 | HECTD4 | NOP2 | SGPL1 | ZFR |
| CNGB3 | HECW1 | NOS1 | SH2D3A | ZMYM4 |
| CNKSR2 | HECW2 | NOS2 | SH3BGR | ZNF143 |
| CNOT3 | HGF | NOTCH1 | SH3PXD2A | ZNF350 |
| CNOT4 | HID1 | NOTCH2 | SHISA4 | ZNF385A |
| CNTN1 | HIST1H3B | NOTCH3 | SI | ZNF414 |
| CNTN4 | HLA-DRB1 | NOTCH4 | SIDT2 | ZNF512B |
| CNTN5 | HLA-DRB5 | NRAS | SIK3 | ZNF541 |
| CNTNAP3B | HMCN1 | NRXN1 | SIM1 | ZNF563 |
| CNTNAP5 | HMHA1 | NRXN2 | SIM2 | ZNF614 |
| COASY | HNF4A | NT5C3L | SLC13A3 | ZNF687 |
| COL14A1 | HOMER2 | NTM | SLC17A6 | ZNF705B |
| COL16A1 | HPS3 | NTRK1 | SLC17A8 | ZNF705G |
| COL19A1 | HPS4 | NTRK3 | SLC25A1 | ZNF711 |
| COL1A1 | HRAS | NUDCD2 | SLC25A30 | ZNF804B |
| COL25A1 | HSPA12B | NUP205 | SLC26A3 | ZSWIM8 |
| COL4A5 | COL4A6 | COL5A1 | COL5A2 | COL5A3 |
| COL6A5 | COL6A6 | COL9A1 | COPA | COPG1 |
| CPA1 | CPSF3 | CPSF6 | CRKL | CRTAM |
| CRTAP | CRYBG3 | CSF1R | CSMD1 | CSMD3 |
| CSN3 |  |  |  |  |
