# Supplementary material for: S6K1 amplification confers innate resistance to CDK4/6 inhibitors through activating c-Myc pathway in patients with estrogen receptor-positive breast cancer
Source: Mol Cancer. 2022 Aug 30;21:171. doi: 10.1186/s12943-022-01642-5 (PMC9426012; doi:10.1186/s12943-022-01642-5)
Supplement: Supplementary file 3 — Additional file 3: Supplementary Table S3. Baseline clinical and pathological characteristics of patients in the discovery cohort. [file 12943_2022_1642_MOESM3_ESM.docx]

Supplementary Table S3. Baseline clinical and pathological characteristics of patients in the discovery cohort.

|  | All^#^  N=36 | Innate resistance  N=20 | Clinical benefit  N=15 | *P^*^* |
| --- | --- | --- | --- | --- |
| Age, n (%)  <40  40-69  >70 | 9 (25.0%)  25 (69.4%)  2 (5.6%) | 6 (30.0%)  13 (65.0%)  1 (5.0%) | 2 (13.3%)  12 (80.0%)  1 (6.7%) | 0.609 |
| Stage at diagnosis, n (%)  I  II  III  IV | 5 (13.9%)  19 (52.8%)  3 (8.3%)  9 (25.0%) | 3 (15.0%)  10 (50.0%)  3 (15.0%)  4 (20.0%) | 2 (13.3%)  9 (60.0%)  0  4 (26.7%) | 0.534 |
| ER/PR status  ER+PR-  ER+PR+  ER-PR+ | 12 (33.3%)  23 (63.9%)  1 (2.8%) | 4 (20.0%)  15 (75.0%)  1 (5.0%) | 8 (53.3%)  7 (46.7%)  0 | 0.071 |
| Visceral metastasis  Yes  No | 27 (75.0%)  9 (25.0%) | 16 (80%)  4 (20.0%) | 10 (66.7%)  5 (33.3%) | 0.451 |
| Bone-only metastasis  Yes  No | 1 (2.8%)  35 (97.2%) | 0  20 (100.0%) | 1 (6.7%)  14 (93.3%) | 0.429 |
| Combined endocrine therapy  Fulvestrant  Letrozole  Exemestane  Anastrozole | 18 (50.0%)  10 (27.8%)  5 (13.9%)  3 (8.3%) | 11 (55.0%)  4 (20.0%)  3 (15.0%)  2 (10.0%) | 6 (40.0%)  6 (40.0%)  2 (13.3%)  1 (6.7%) | 0.665 |
| Previous lines of therapy, N (%)  0-1  2-4  >5 | 8 (22.2%)  26 (72.2%)  2 (5.6%) | 4 (20.0%)  15 (75.0%)  1 (5.0%) | 4 (26.7%)  10 (66.7%)  1 (6.7%) | 0.864 |
| Documented sensitivity to prior hormone therapy  Yes  No | 23 (63.9%)  13 (36.1%) | 12 (60.0%)  8 (40.0%) | 10 (66.7%)  5 (33.3%) | 0.737 |
| ^#^one out of 36 patients interrupted treatment due to toxicity, thus was not included in the subsequent data sets for efficacy evaluation  *compared between patients with innate resistance and those benefit from CDK4/6 inhibitor  ER, estrogen receptors; PR, progesterone receptors. | | | | |
