# Supplementary figures and images for: S6K1 amplification confers innate resistance to CDK4/6 inhibitors through activating c-Myc pathway in patients with estrogen receptor-positive breast cancer
Source: Mol Cancer. 2022 Aug 30;21:171. doi: 10.1186/s12943-022-01642-5 (PMC9426012; doi:10.1186/s12943-022-01642-5)

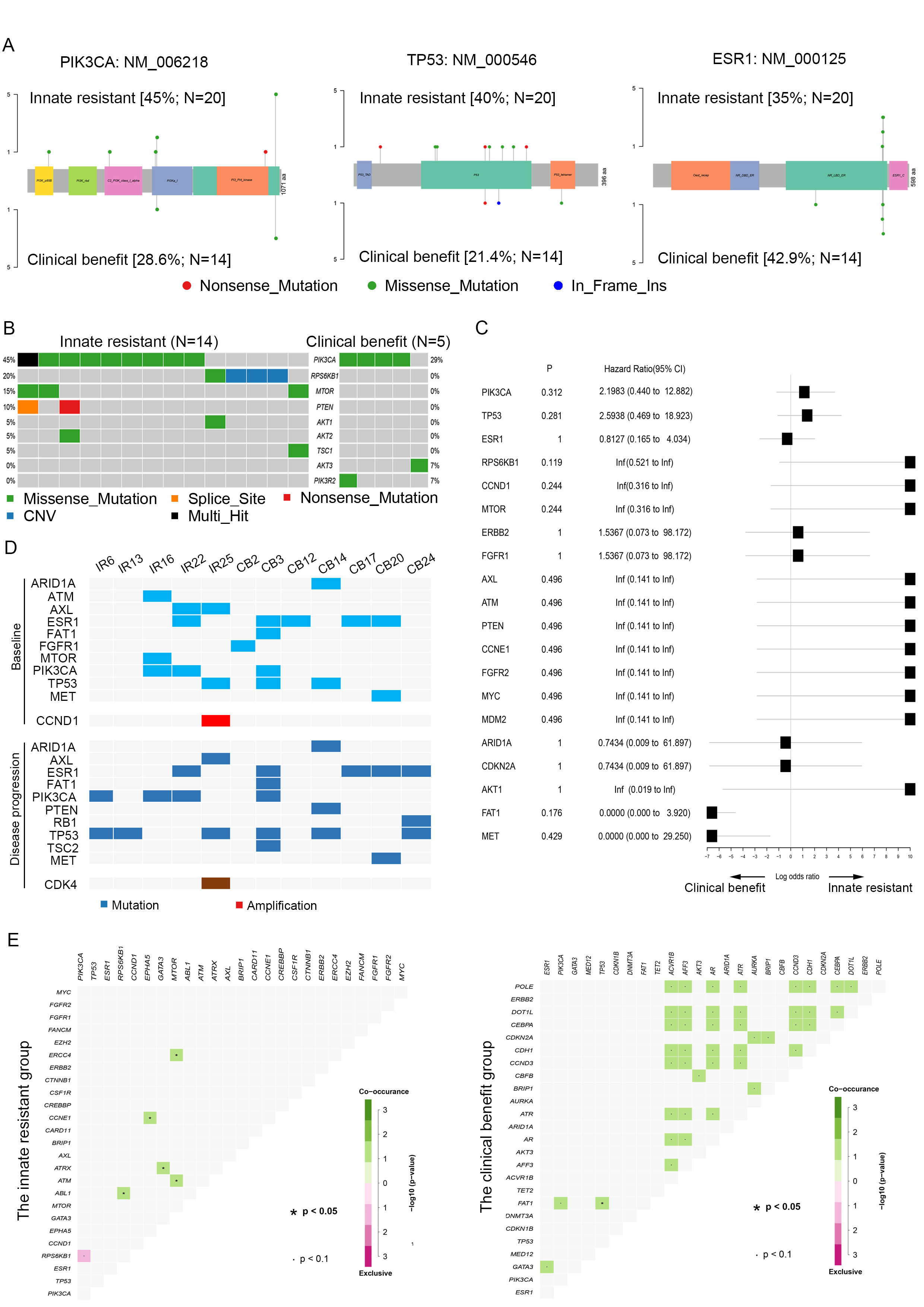

Supplement: Supplementary file 8 — Additional file 8: Supplementary Fig. S1. Analysis of genetic abnormalities in plasma samples from patients receiving palbociclib treatment in the discovery cohort (related to Fig. 1). (A) The mutated locus of PIK3CA, TP53, and ESR1 genes in patients with or without innate resistance to palbociclib. (B) Diverse PI3K-pathway activating events observed in 19 patients. (C) The detected molecular alterations in functional genes of patients with or without innate resistance to palbociclib. (D) The detected molecular alterations at the baseline and after disease progression. IR: innate resistant; CB: clinical benefit. (E) The analysis of interactions between high-frequency altered genes in patients with or without innate resistance to palbociclib. S6K1 appears to be mutually exclusive with PIK3CA gene in patients with innate resistance to palbociclib. [file 12943_2022_1642_MOESM8_ESM.tif]

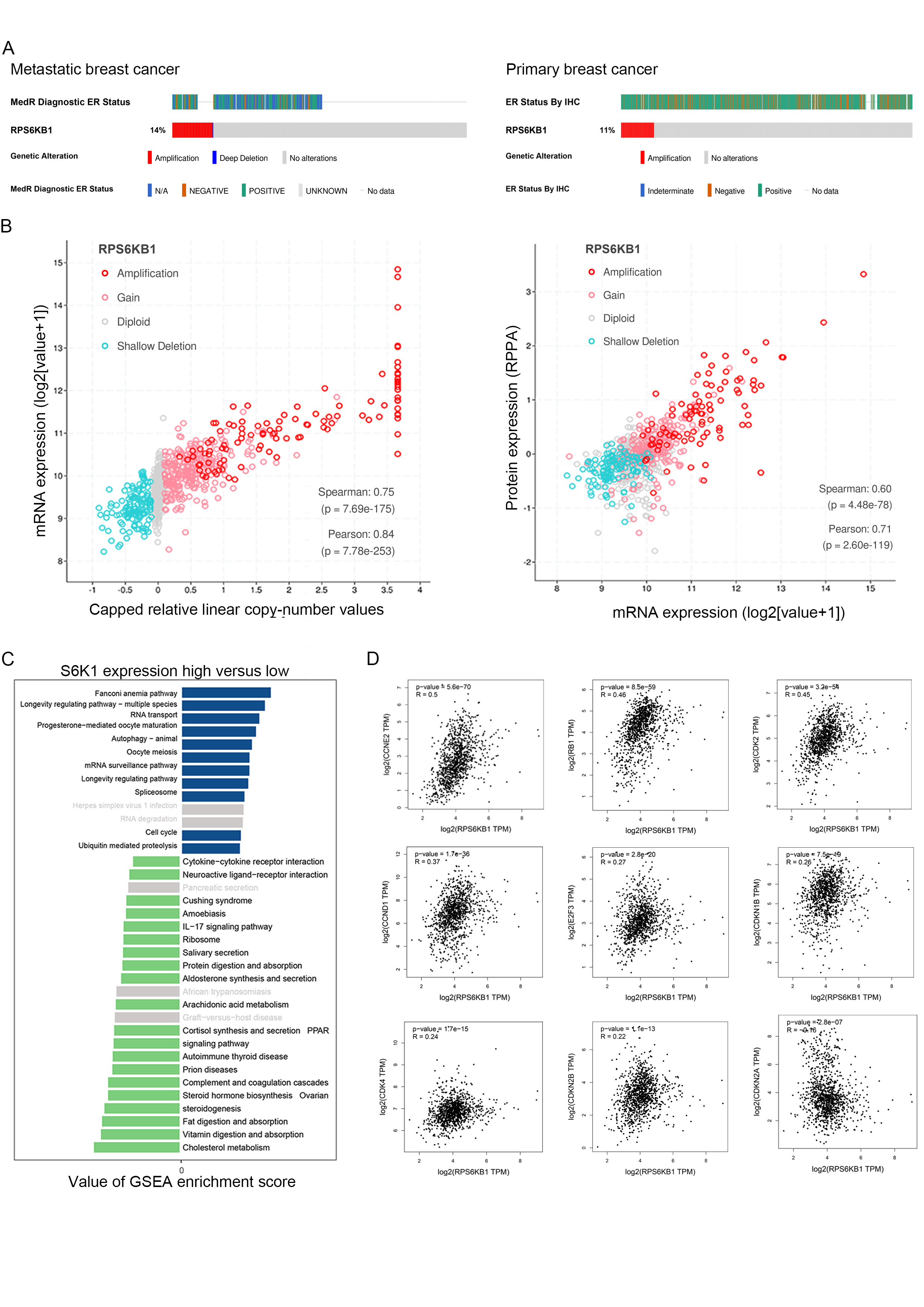

Supplement: Supplementary file 9 — Additional file 9: Supplementary Fig. S2. S6K1 gene amplification is correlated with high expression of S6K1 and cell cycle-related genes in the TCGA cohort (related to Fig. 2). (A) Copy number variation of S6K1 (RPS6KB1) gene in patients with metastatic or primary breast cancer, based on cBioPortal. (B) Analysis of correlations between S6K1 (RPS6KB1) gene amplification and S6K1 mRNA expression (960 samples with data in both profiles) as well as S6K1 mRNA and protein expression (780 samples with data in both profiles) in breast cancer, based on cBioPortal. (C) The signalling pathways that are affected by high expression of S6K1 analysed by GSEA. (D) Analysis of correlations between S6K1 and cell cycle-related gene expression in BRCA cohort, based on GEPIA. P value was calculated by Spearman’s correlation. [file 12943_2022_1642_MOESM9_ESM.tif]

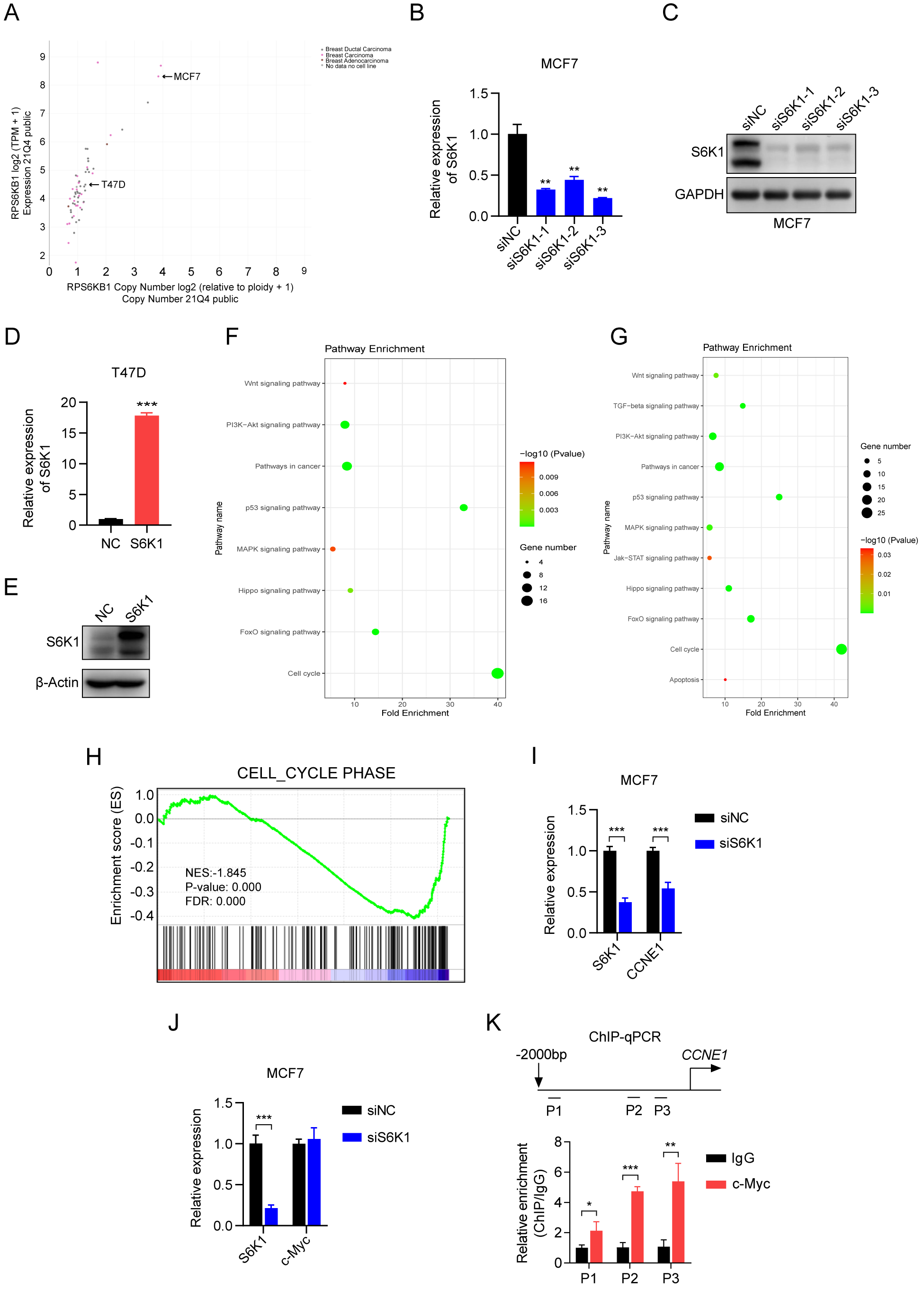

Supplement: Supplementary file 10 — Additional file 10: Supplementary Fig. S3. Related to Figs. 4 and 5. (A) The copy number and mRNA levels of S6K1 in breast cancer cell lines were obtained from Cancer Cell Line Encyclopedia (CCLE) (https://portals.broadinstitute.org/ccle). Y-axis represents mRNA expression and X-axis represents copy number. (B, C) MCF-7 cells were transfected with S6K1 siRNAs (siS6K1-1/− 2/− 3) or non-sense control siRNA (siNC). The RNAi efficiency was validated using RT-qPCR (B) and western blotting (C). (D, E) Stable exogenous S6K1-expressed T47D cells and control cells were validated using RT-qPCR (D) and western blotting (E). (F, G) KEGG enrichment of signalling pathways of phosphorylated (F) or non-phosphorylated (G) proteins detected by protein chip in S6K1-depleted MCF-7 cells. (H) GSEA plot of RNA-seq data from S6K1-depleted MCF-7 cells for cell cycle phase. (I, J) MCF-7 cells were transfected with S6K1 siRNA pool or non-sense control siRNA. The mRNA levels of c-Myc and cyclin E1 were measured using RT-qPCR. (K) ChIP assay for the binding ability of c-Myc to the cyclin E1 promoter element in MCF-7 cells. P value was calculated by Student’s t-test. *, P < 0.05, **, P < 0.01, ***, P < 0.001. [file 12943_2022_1642_MOESM10_ESM.tif]

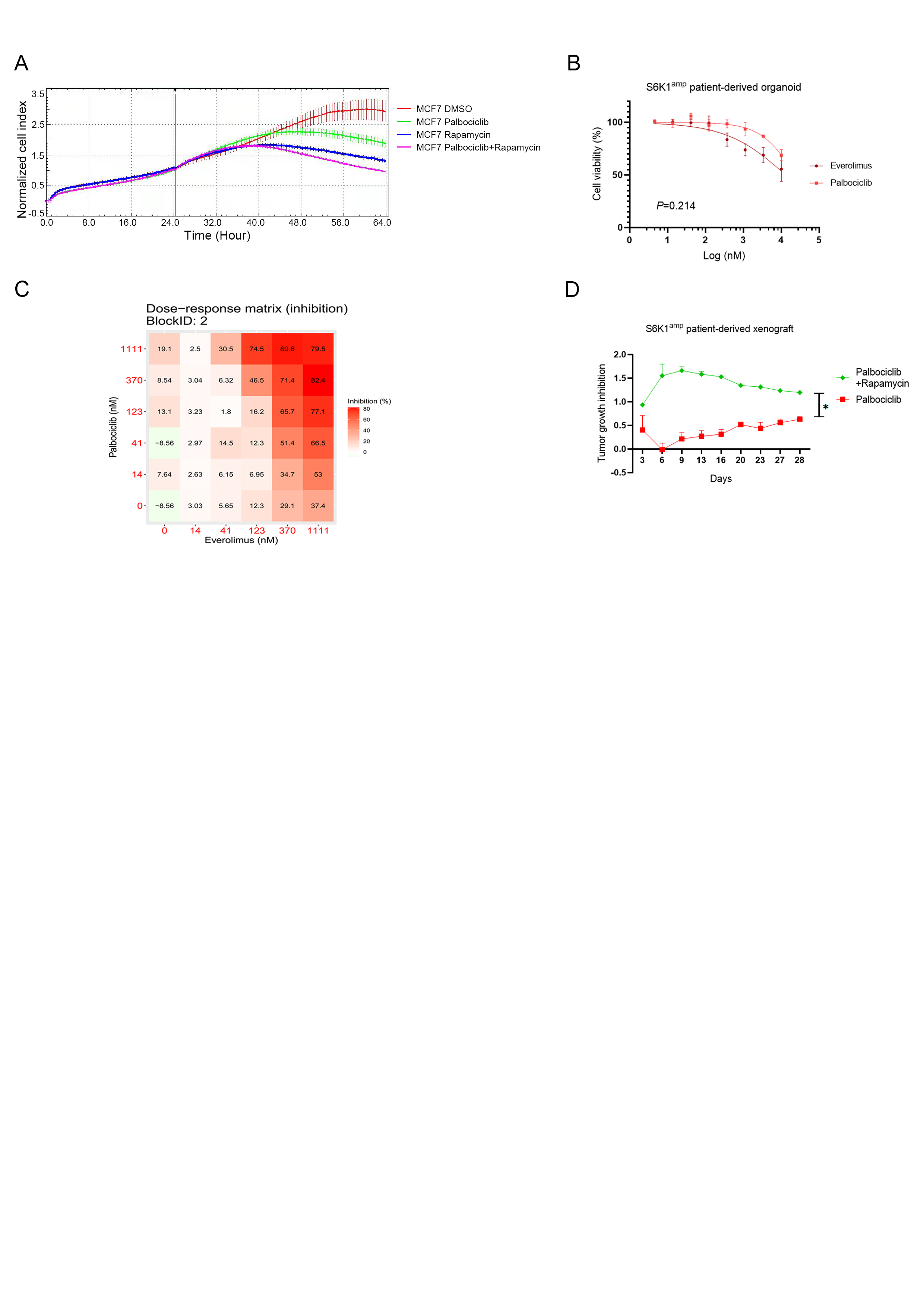

Supplement: Supplementary file 11 — Additional file 11: Supplementary Fig. S4. Related to Fig. 6. (A) xCELLigence system analysis of the proliferation of MCF-7 cells treated with either vehicle, palbociclib (5 μM), rapamycin (1 μM) or combination. (B) S6K1-amplified PDOs from breast cancers showed limited response to everolimus or palbociclib as monotherapy. Data are presented as the mean ± SEM; P value was calculated by 2-way ANOVA. (C) Dose–response matrix for the effect of palbociclib plus mTOR inhibitor (everolimus) in a S6K1-amplificated organoid derived from breast cancer patient. (D) Tumour growth inhibition (TGI) curve in S6K1-amplificated xenograft derived from breast cancer patient. Data are presented as the mean ± SEM; P value was calculated by Mixed-effects model. *, P < 0.05. TGI was calculated using the following formula: TGI = 1 − (tumour volume change of the treated group relative to day 0)/(tumour volume change of the control group relative to day 0). [file 12943_2022_1642_MOESM11_ESM.tif]
